# Differential Action of Reelin on Oligomerization of ApoER2 and VLDL Receptor in HEK293 Cells Assessed by Time-Resolved Anisotropy and Fluorescence Lifetime Imaging Microscopy

**DOI:** 10.3389/fnmol.2019.00053

**Published:** 2019-02-26

**Authors:** Paula Dlugosz, Roland Tresky, Johannes Nimpf

**Affiliations:** Department of Medical Biochemistry, Max F. Perutz Laboratories, Medical University Vienna, Vienna, Austria

**Keywords:** ApoER2, VLDLR, receptor clustering, FRET, FLIM, anisotropy, reelin

## Abstract

The canonical Reelin signaling cascade regulates correct neuronal layering during embryonic brain development. Details of this pathway are still not fully understood since the participating components are highly variable and create a complex mixture of interacting molecules. Reelin is proteolytically processed resulting in five different fragments some of which carrying the binding site for two different but highly homologous receptors, apolipoprotein E receptor 2 (ApoER2) and very low density lipoprotein receptor (VLDLR). The receptors are expressed in different variants in different areas of the developing brain. Binding of Reelin and its central fragment to the receptors results in phosphorylation of the intracellular adapter disabled-1 (Dab1) in neurons. Here, we studied the changes of the arrangement of the receptors upon Reelin binding and its central fragment at the molecular level in human embryonic kidney 293 (HEK293) cells by time-resolved anisotropy and fluorescence lifetime imaging microscopy (FLIM). In the off-state of the pathway ApoER2 and VLDLR form homo or hetero-di/oligomers. Upon binding of full length Reelin ApoER2 and VLDLR homo-oligomers are rearranged to higher order receptor clusters which leads to Dab1 phosphorylation. When the central fragment of Reelin binds to the receptors the cluster size of homo-oligomers is not affected and Dab1 is not phosphorylated. Hetero-oligomerization, however, can be induced, but does not lead to Dab1 phosphorylation. Cells expressing only ApoER2 or VLDLR change their shape when stimulated with the central fragment. Cells expressing ApoER2 produce filopodia/lamellipodia and cell size increases, whereas VLDLR-expressing cells decrease in size. These findings demonstrate that the primary event in the canonical Reelin pathway is the rearrangement of preformed receptor homo-oligomers to higher order clusters. In addition the possibility of yet another signaling mechanism which is mediated by the central Reelin fragment independent of Dab1 phosphorylation became apparent.

## Introduction

Development of the multilayered structure of the mammalian forebrain is mainly orchestrated by Reelin which is secreted by Cajal-Retzius cells in the marginal zone (D’Arcangelo et al., 1995; Curran and D’Arcangelo, 1998). Disruption of the Reelin pathway leads to the well described “reeler phenotype” which is characterized by the name giving motor deficits and an abnormal layering of laminated structures in the cortex, hippocampus, and cerebellum (reviewed in Tissir and Goffinet, 2003; Frotscher, 2010). Reelin also shapes synapses and modulates their function (Herz and Chen, 2006; Wasser and Herz, 2017). Correct layering of the cortex during embryonic brain development is mediated by the canonical Reelin-signaling pathway which starts with the binding of Reelin to apolipoprotein E receptor 2 (ApoER2) and/or very low density lipoprotein receptor (VLDLR) as recently reviewed in Bock and May (2016) and Santana and Marzolo (2017). The most critical event in this pathway is the Src-family tyrosine kinase mediated phosphorylation of the intracellular adapter protein Dab1. ApoER2 and VLDLR belong to a large family of receptors related to the low-density lipoprotein (LDL) receptor the main function of which is to mediate endocytosis of LDL (reviewed in Herz and Bock, 2002). Whether co-receptors or alternate receptors are required to induce the “core-event” of the pathway i.e., Dab1 phosphorylation is still under debate. The current consensus-concept of the signaling pathway postulates that Reelin or proteolytic fragments thereof form dimers or higher order multimers (Kubo et al., 2002; Jossin et al., 2004; Yasui et al., 2011) which upon binding cluster the receptors and as a consequence induce di- or multimerization of Dab1 (Strasser et al., 2004; Divekar et al., 2014). Signaling events downstream of Dab1 are manifold including PI3K- and Rap1-activation, n-cofilin phosphorylation, and Cullin5 mediated Dab1 degradation (Bock and May, 2016). The actual initiation of the cascade, i.e., phosphorylation of Dab1 seems to be straight forward but its detailed mechanism is still not well understood. First of all, the suggested clustering of the receptors by their ligand(s) was never directly demonstrated on live cells. Second, the story is fairly complex since at least two different receptors i.e., ApoER2 and VLDLR are involved. This is demonstrated by genetic experiments showing that only the double knock out of both receptors recapitulates the full “reeler phenotype” (Trommsdorff et al., 1999), whereas loss of either ApoER2 or VLDLR alone produces distinctive phenotypes on their own. Thus, the same pathway mediated by either ApoER2 or VLDLR has distinctive physiological functions during brain development (see below and reviewed and discussed in Chai and Frotscher, 2016; Kon et al., 2017). In addition, this situation is even more complex taking the following observations into account. One is that ApoER2 and VLDLR are expressed in distinct spatiotemporal patterns in the developing cortex (Uchida et al., 2009; Hirota et al., 2015). The second is that distinct variants of ApoER2 exist which are produced by differential splicing (Clatworthy et al., 1999) and/or differential glycosylation (May et al., 2003; Wasser et al., 2014) and which reside in distinct subdomains of the cell membrane (Duit et al., 2010). The third lays in the complex structure of Reelin which is proteolytically cleaved at three sites (Lee and D’Arcangelo, 2016) producing five major fragments which exhibit different abilities to diffuse in brain tissue (Lambert de Rouvroit et al., 1999; Jossin et al., 2004; Jossin et al., 2007).

During development of the cortex neurons undergo several changes in polarity on their way from their birth place to their final position in the mature organ (reviewed in Kon et al., 2017). At the beginning of cortical development polarized neuronal precursors, which are derived from asymmetric division of radial glia cells, migrate *via* somal translocation to their final destination. As soon as the cortex becomes too thick for such a movement these precursors switch to a multi-phase mode of migration. They leave the ventricular zone by bipolar migration, lose their polarity, and switch to a multipolar migration mode establishing a specific region of the intermediate zone the so called “multipolar morphology zone” (MMZ). Then, the cells switch again to a bipolar migration mode guided be radial glia and establish the cortical plate by terminal translocation (Nadarajah et al., 2001).

How is this complex migratory pattern orchestrated by Reelin? Based upon a significant body of evidence from genetic and cell biological experiments and taking into account the spatiotemporal expression of ApoER2 and VLDLR during this process (Hirota et al., 2015), an intricate model was suggested (Chai and Frotscher, 2016; Frotscher et al., 2017). The key actions of Reelin therein are to induce re-polarization of multipolar cells in the intermediate zone by regulating expression of focal adhesion molecules and stabilizing the leading process along the radial fiber. This action seems to be mediated by ApoER2. In the marginal zone, however, Reelin stops over-migration primarily by interaction with VLDLR.

The aim of this study was to investigate whether the initial event of the Reelin signaling cascade differs whether ApoER2 or VLDLR is involved. Reelin-induced clustering of ApoER2 and VLDLR was analyzed using time-resolved anisotropy (homo-FRET; Förster resonance energy transfer) for homo-oligomerization and fluorescence lifetime imaging microscopy (FLIM-FRET) for hetero-oligomerization of the receptors.

## Materials and Methods

### Animals

Sprague-Dawley rats were purchased from the Biomedical Research Division for Laboratory Animals, Medical University of Vienna. Animal handling and sacrificing were approved by the Austrian Federal Ministry of Science and Research (permit number, BMWFW-66.006/0012-WF/II/3b/2014) and were undertaken in strict accordance with prevailing guidelines for animal care and welfare.

### Reagents and Antibodies

iDimerize™ Inducible Homodimer System containing pHom1 and pHomMem1 plasmids and Homodimerizer (AP20187) were purchased from Clontech. Fluorescein (F2456) was from Sigma Aldrich. Central Reelin fragment (3820-MR-025) was from bio-techne. Restriction enzymes and T4 Ligase were from Thermo Scientific. Q5 High-Fidelity DNA Polymerase was from New England Biolabs. Antibodies used in this study are summarized in Table 1.

**Table 1:** The following antibodies were used in this study at the indicated dilutions.

Epitope	Catalog number	Company/Reference	Dilution
VLDLR	AF2258	R&D Systems	WB 1:2,000
VLDLR (6A6)	sc-18824	Santa Cruz Biotechnology	IP
VLDLR	Ab74	Strasser et al. (2004)	WB 1:2,000
Reelin	Clone G10, MAB5364	EMD Milipore	WB 1:15,000, IF 1:1,000
(CGY)FP	AB121	evrogen	WB 1:8,000
mCherry	1C51, NBP1–96752	Novus Biologicals	WB 1:1,000
GAPDH	#G8795	Sigma Aldrich	WB 1:10,000
Phospho-Dab1 (Tyr232)	#3325	Cell Signaling	WB 1:1,000
Dab1	D4	a kind gift from André Goffinet, University of Louvain, Belgium	WB 1:8,000
ApoER2	Ab108208	abcam	WB 1:1,000
ApoER2	Ab186	Strasser et al. (2004)	IP
ApoER2	Ab20	Stockinger et al. (1998)	WB 1:10,000
HA	Clone 3F10, #11867423 001	Roche	IP
Normal mouse IgG	sc-2025	Santa Cruz Biotechnology	IP
Normal rabbit IgG	#12–370	EMD Millipore	IP

### Cloning

Constructs pHom1_mGFP and pHomMem1_mGFP containing monomeric GFP (mutated at position A206K by site-directed mutagenesis; Zacharias et al., 2002) and either one copy of FK506 binding protein (FKBP) binding protein (pHom1) or 2 copies of FKBP and a N-terminal myristoylation signal for membrane localization (pHomMem1) were constructed by PCR amplification of mGFP from pHom1_EGFR_mGFP (Hofman et al., 2010) using the following primers: 5′-atatactagtatggtgagcaagggcgagg-3′ and 5′-atatggatccttacttgtacagctcgtcca-3′, which introduced flanking restriction sites *SpeI* and *BamHI* (underlined). The mGFP PCR product was inserted into the corresponding sites of pHom1 and pHomMem1 to produce pHom1_mGFP and pHomMem1_mGFP. To construct pmGFP the FKBP domain from pHom1_mGFP was removed by digestion with *XbaI* and *SpeI* and self-ligation.

To generate pHomMem1_mCherry (containing two copies of FKBP and mCherry at the C-terminal), the cDNA coding for mCherry was amplified by PCR from pmCherry-N1 (Clontech) using the following primers 5′-atatactagtatggtgagcaagggcgagg-3′ and 5′-atatggatccttacttgtacagctcgtcca-3′, which introduced flanking restriction sites *SpeI* and *BamHI* (underlined). The mCherry PCR product was inserted into the corresponding sites of pHomMem1 to produce pHomMem1_mCherry.

To generate pHom1_VLDLR_mGFP and pHomMem1_VLDLR_mGFP, the cDNA for VLDLR was amplified by PCR from pClneo_VLDLR (murine VLDLR lacking the O-linked sugar domain, which is the predominant splice form in murine brain; Mayer et al., 2006) using primers 5′-atatgaattcatgggcacgtccgcgcgct-3′ and 5′-atattctagaagccagatcatcatctgtgc-3′ and was inserted into pHom1_mGFP and pHomMem1_mGFP digested with *EcoRI* and *XbaI*.

To generate pHom1_ApoER2_mGFP and pHomMem1_ApoER2_mGFP ApoER2 was amplified by PCR from pClneo_ApoER2 (Koch et al., 2002) using primers 5′-atatgaattcatgggccgcccagaactg-3′ and 5′-atattctagagggcagtccatcatcttcaa-3′ and was inserted into pHom1_mGFP and pHomMem1_mGFP digested with *EcoRI* and *XbaI*. To construct pVLDLR_mGFP and pApoER2_mGFP, the FKBP domain from pHom1_VLDLR_mGFP and pHom1_ApoER2_mGFP was removed by digestion with *XbaI* and *SpeI* and self-ligation.

pmCherry-N1_ApoER2 was cloned by ligating the cDNA for mmApoER2 into pmCherry-N1 which was digested with *SalI* and *HindIII* using T4 ligase. pcDNAflux3_ApoER2_HA plasmid was constructed as described in Hoe and Rebeck (2005).

pHom1_EGFR_mGFP, pHomMem1_EGFR_mGFP was a kind gift from Paul M. P. van Bergen en Henegouwen (Utrecht University). pSetB_GFP was a kind gift from Ivan Yudushkin (Max F. Perutz Laboratories, Vienna). pGFP_mCherry (hetero-FRET positive control) was a kind gift from Dea Slade (Max F. Perutz Laboratories, Vienna). pcDNA5myc_Dab1 was a kind gift from Peggy Stolt-Bergner (Protein Technologies Facility, VBCF). Fidelity of all constructs was tested by sequencing.

### Cell Lines and Transfection

Human embryonic kidney cells 293 (HEK293, ATCC) were cultivated in Dulbecco’s modified Eagle’s medium (DMEM; Gibco) supplemented with 10% fetal calf serum (Sigma, St. Louis, MO, USA) at 37°C and 5% CO_2_. Stable NIH3T3 (mouse embryonic fibroblasts) VD line expressing murine VLDLR lacking the O-linked sugar domain and murine Dab1 (Mayer et al., 2006) were kept under puromycin selection (2 μg/ml). The cell lines were tested for mycoplasma contamination using PlasmoTest (InvivoGen, San Diego, CA, USA). After 24 h cultivation, cells were transfected using PEI (polyethylenimine) and the indicated constructs according to Tom et al. (2008) incubated for 24 h, followed by imaging, and fixation or cell lysis.

### Preparation of Cell and Tissue Extracts, SDS-PAGE, Western Blotting, Immunoprecipitation

Cell and tissue extracts were prepared in NP-40 lysis buffer (150 mM sodium chloride, 1.0% Nonidet P-40, 10% glycerol, 20 mM Tris, pH 7.4) supplemented with cOmplete™ EDTA-free Protease Inhibitor Cocktail (Roche) and 1 mM EDTA and used directly for SDS-PAGE or for immunoprecipitation. Extracts were precleared and then incubated overnight at 4°C with anti-ApoER2 (Ab186), anti-VLDLR (Ab6A6), and anti-GFP (Ab(CGY)FP) crosslinked to Protein A Sepharose 4B (Invitrogen, Carlsbad, CA, USA). Beads were collected by centrifugation at 500× *g* for 1 min and washed three times using NP-40 lysis buffer supplemented with Protease Inhibitor Cocktail and 1 mM EDTA. Bound proteins were eluted twice with 100 mM glycine pH 2.5 by rotating the sample for 10 min at room temperature. The eluates were collected after 1 min centrifugation at 3,000× *g* and immediately neutralized by adding 5–7 μl 1 M Tris-HCl pH 9. 4× SDS Protein Sample Buffer was added to the eluates and the samples were boiled for 10 min at 95°C. Proteins were separated by reducing SDS-PAGE and transferred onto Amersham™ Protran nitrocellulose membrane (GE Healthcare, Chicago, IL, USA) by wet blotting. Membranes were blocked in PBS containing 0.1% Tween-20 and 5% bovine serum albumin (BSA) or 5% milk and incubated with primary antibody over night at 4°C. After washing membranes were incubated with HRP-conjugated secondary antibodies (Jackson ImmunoResearch Laboratories, West Grove, PA, USA). For detection, enhanced chemiluminescence solution NOVA 2.0 (Cyanagen) was used.

### Pulse Chase of ^35^S Methionine, Cysteine Labeled ApoER2

HEK293 cells were seeded on 35 mm dishes and allowed to grow to a confluency of 80% at 37°C, 5% CO_2._ Cells were transfected with pcDNAflux3_ApoER2_HA. After 24 h cells were shifted to Dulbecco’s Modified Eagle’s Medium without L-methionine and L-cysteine supplemented with 10% fetal calf serum for 30 min. Then, cells were labeled with 200 μCi/mL EasyTag™ EXPRESS^35^S Protein Labeling Mix (PerkinElmer) for 30 min (pulse period) followed by the chase period for the indicated time intervals in Dulbecco’s modified Eagle’s medium supplemented with 10% fetal calf serum. After the chase period, the cells were washed twice with 2 ml of ice-cold PBS and then lysed by addition of 0.3 ml of NP-40 lysis buffer supplemented with cOmplete™ EDTA-free Protease Inhibitor Cocktail and 1 mM EDTA. ApoER2_HA was immunoprecipitated from the lysates using anti-HA plus Protein G Sepharose (Invitrogen, Carlsbad, CA, USA) over night at 4°C. The immunoprecipitates were dissolved by heating at 95°C for 10 min in 4× SDS Protein Sample Buffer and then subjected to electrophoresis on 8% or 5% SDS-PAGE followed by either fixation, treatment with EN3HANCE solution (PerkinElmer, Waltham, MA, USA), and drying for autoradiography, or processed for western blotting using Ab20 as described above.

### Dab1 Phosphorylation Assay

HEK293 cells were grown on 12-well plates coated with 50 μg/mL OptiCol™ human collagen type I (Cell Guidance Systems) to a confluency of 80%. Cells were transfected with pcDNA5myc_Dab1 and pClneo_ApoER2 or pcDNA5myc_Dab1 and pClneo_VLDLR or pcDNA5myc_Dab1, pClneo_ApoER2, and pClneo_VLDLR. After 24 h, cells were washed with PBS and kept in Imaging Medium (Hank’s Balanced Salt Solution, 2 mM Glutamine, 10 mM HEPES) for 30 min. Next, medium was changed to Reelin conditioned medium (RCM) or R3–6 was added to the cells (final concentration, 30 nM). After 20 min incubation at 37°C, cells were washed with cold PBS and lysed in NP-40 lysis buffer (150 mM sodium chloride, 1.0% Nonidet P-40, 10% glycerol, 20 mM Tris, pH 7.4) supplemented with cOmplete™ EDTA-free Protease Inhibitor Cocktail (Roche), 1 mM EDTA, 0.05 M NaF and 1 mM Na_3_VO_4_. The lysates were centrifuged at 15,000× *g* for 15 min at 4°C. Protein concentration was measured with BCA Protein Assay Kit (Thermo Scientific) and 30 μg of proteins were separated on a 10% SDS-PAGE and immunoblotted for phospho-Dab1 (Ab pDab1, Cell Signaling) or GAPDH (Sigma Aldrich, St. Louis, MO, USA). Membranes were stripped and subsequently re-probed for the detection of Dab1 (AbD4).

### Immunofluorescence

HEK293 cells were grown on coverslips in 24-well plates coated with 50 μg/mL OptiCol™ human collagen type I (Cell Guidance Systems) to a confluency of 50–70%. Cells were transfected with pVLDLR_mGFP, pApoER2_mGFP, pmCherry-N1_ApoER2 + pVLDLR_mGFP or mGFP. After 24 h, 25 μl of concentrated RCM (cRCM) was added to the wells. Cells were incubated with cRCM at 37°C for 5 min, washed twice with cold PBS and fixed with 4% formaldehyde for 15 min at room temperature. Fixed cells were washed three times with cold PBS, incubated in blocking solution (1% BSA in PBS) for 30 min at room temperature and overnight with primary antibody (anti-Reelin, G10) at 4°C. On the next day, samples were washed three times with cold PBS and incubated with secondary antibody, goat anti-mouse IgG DyLight633 (ThermoScientific) for 1 h at RT. Afterwards, cells were washed three times with cold PBS and incubated 5 min in DAPI solution (5 μg/ml), washed again, incubated in quenching buffer (100 mM glycine) for 15 min. After the final wash with H_2_0, coverslips were mounted using ibidi Mouting Medium (ibidi) and sealed with nail polish. Slides were analyzed using a confocal fluorescence microscope (laser-scanning microscope 700, Zeiss) and the corresponding ZEN software.

### Preparation of Reelin Conditioned Medium

Reelin-expressing HEK293 cells were cultivated and used for production of RCM as described before (Brandes et al., 2001). Briefly, HEK293 cells stably carrying the full-length mouse Reelin expression construct pCrl (a kind gift of Tom Curran, Perelman School of Medicine at the University of Pennsylvania, Philadelphia, PA, USA) were cultivated in DMEM supplemented with 10% fetal calf serum (Invitrogen, Carlsbad, CA, USA), and 0.2 mg/ml G418 at 37°C and 5% CO_2_. When the cells reached 70% confluency the culture medium was replaced by serum-free medium (OptiMEM, Gibco). After two more days the conditioned medium was collected, sterile filtered, and concentrated by ultra-centrifugation overnight (cRCM). The resulting pellet was dissolved and stored at −80°C until use.

### Preparation of GFP_His

GFP_His was purified from *E.coli* culture by Ni-NTA agarose (Qiagen) chromatography under native condition according to the manufacturer’s protocol. Expression of GFP_His was confirmed by Coomassie staining and western blotting. Buffer exchange (to PBS) was performed by dialysis.

### Homo-, Hetero-FRET Measurements

Time-resolved fluorescence anisotropy imaging (TR-FAIM) and FLIM was carried out on an objective scanning confocal inverted microscope setup (Microtime 200, Picoquant, Germany) using a 60× 1.20 NA water immersion objective (Olympus UPlanSApo). Briefly, collimated linearly polarized excitation light from a 485 nm pulsed laser diode (40 MHz repetition rate) was coupled into the optical path *via* a dichroic mirror (Di-R488-25x36, Semrock) and over-filled the back aperture of the objective lens. On the detection arm, light was first focused through a 75 μm pinhole. For TR-FAIM the collimated light was then divided into parallel and perpendicular polarization components using a broadband polarizing beam-splitter, passed through interference bandpass filters (FF01-525/45, Semrock) and focused onto respective hybrid PMA detectors. For FLIM measurements the collimated light was spectrally split using a dichroic mirror, passed through interference bandpass filter (525/45) and focused onto the respective hybrid PMA detector. Images were recorded in a 128 × 128 pixel mode with 64 ps time-correlated single photon counting (TCSPC) resolution, 2 ms dwell time.

The instrument response function (IRF) was measured using back scattering of excitation light and inserting the OD 3.0 optical density filter in the excitation path. In order to obtain IRF perpendicular, polarization of excitation light was rotated using a half wave plate (10RP52-1/λ/2vis, ThorLabs) until the peak intensity in the perpendicular channel was reached and measured as described above. Single photon counting was performed using the TCSPC PicoHarp 300 Module (Picoquant, Germany), and analyzed using FLIMfit software packages (Imperial College London).

To analyze hetero-oligomerization, VLDLR_mGFP fluorescence decay profiles were fitted pixelwise to a bi-exponential decay model. The maximum likelihood fitting algorithm was used for data analysis (Warren et al., 2013, 2015). Goodness of the fit was judged by *χ*^2^-test which should be around 1 and residuals should be evenly distributed across the full extent of the data range.

Obtained fluorophore lifetimes (tau1 and tau2) and lifetime contributions (beta1 and beta2) were then fixed and used in global data analysis. FRET efficiency (E) was determined by fitting the FRET positive control (GFP_mCherry) and was constrained to 0.5 and gamma 1 values (FRET contributions with fixed E) were determined. In each measurement the amount of acquired photons was above 10,000 counts in the maximum. Pixel intensity threshold was set up for 100 photons. Bleaching was evaluated in all time course experiments and any data points in which bleaching reached or exceeded 20% of initial intensity were subtracted from data analysis. Cells that were acquired with high excitation laser power and could be influenced by pile-up effect were also subtracted from data analysis.

To analyze homo-oligomerization, time-resolved anisotropy data were analyzed by global approach where a bi-exponential fluorescence decay and bi-exponential anisotropy decay model was used. Goodness of the fit was judged by *χ*^2^-test which should be around 1 and residuals should be evenly distributed across the full extent of the data range. The G-factor (G) was measured using 10 μM fluorescein. Cells were seeded on 8-well Lab-Tek Chambered coverglass (Thermo Scientific) coated with collagen (50 μg/mL) and after 24 h cells were transfected with the indicated plasmids. FRET measurements were performed 24 h after transfection. One hour before measurement DMEM medium was removed, cells were washed with PBS and Imaging Medium (Hank’s Balanced Salt Solution, 2 mM Glutamine, 10 mM HEPES) was added to each well.

### Cell Morphological Analysis

Cells acquired for homo-FRET and hetero-FRET analysis were evaluated for morphological changes by ImageJ (1.52i., USA). To quantify these changes upon R3–6 treatment, we applied the particle measurement feature to measure the area, perimeter, solidity (the ratio of the cell area and the area of the convex hull of the cell; it indicates how “ruffled” the border of a cell is or how many concave cavities are on the surface) and Feret diameter (the greatest distance between any two points along the cell perimeter) of the cells. Before cell measurements, all images were thresholded using Li Auto Threshold method.

### Statistical Analysis

All statistical tests were performed using GraphPad Prism version 6. Prior to analysis, normal distribution of the data was checked by the D’Agostino-Pearson omnibus normality test. To compare two sample groups before and after the treatment paired, two-tailed Student *t*-test was performed. To compare more than two sample groups one way ANOVA multiple comparison test was used. Results were considered significant when *p* ≤ 0.05.

## Results

Circumstantial evidence suggests that Dab1 phosphorylation triggered by Reelin binding to either ApoER2 or VLDLR is achieved by receptor clustering resulting in dimerization or oligomerization of Dab1 on the inner leaflet of the cell membrane rendering the adapter protein to a substrate for Src-family kinases. This view is primarily supported by the finding that artificial dimerization of Dab1 in the absence of receptors and Reelin is sufficient to induce its phosphorylation and that Dab1 phosphorylation can be induced by artificial ligands for ApoER2 and VLDLR as long as they are able to dimerize the receptors (Strasser et al., 2004). In addition, ligand induced clustering of ApoER2 was demonstrated by co-precipitation experiments (Divekar et al., 2014) and Reelin is secreted as disulfide-linked dimers which form higher order multimers in solution (Utsunomiya-Tate et al., 2000; Yasui et al., 2011). It has to be pointed out, however, that this model was never evaluated directly at the level of the receptors. Here, we used TR-FAIM (Levitt et al., 2009; Chan et al., 2011) and FLIM (Day and Davidson, 2012), which allow to directly visualize and measure protein cluster formation in living cells.

We started the assessment of the receptor state(s) in the basal situation i.e., without the addition of Reelin. To evaluate hetero-oligomerization of ApoER2 and VLDLR in living cells we implemented hetero-FRET FLIM measurements. FRET occurs only when the donor molecule is in close proximity (less than 10 nm) to a suitable acceptor fluorophore. FLIM measures the lifetime of a fluorophore, in other words, the time a fluorophore stays in the excited state before returning to the ground state by emitting a photon. When energy transfer (FRET) occurs between two fluorophores, the lifetime of the donor decreases. Measurement of FRET *via* FLIM has multiple advantages over intensity-based FRET approaches, such as independence of fluorophore concentration, scattering, sample absorption and excitation power. The combination of FLIM and FRET studies allows to determine populations of interacting proteins on a pixel-by-pixel basis in subcellular compartments of living cells (Ishikawa-Ankerhold et al., 2012; Shrestha et al., 2015).

### ApoER2 and VLDL Receptor Form Hetero di/oligomers in the Absence of Reelin

**Figure 1:**
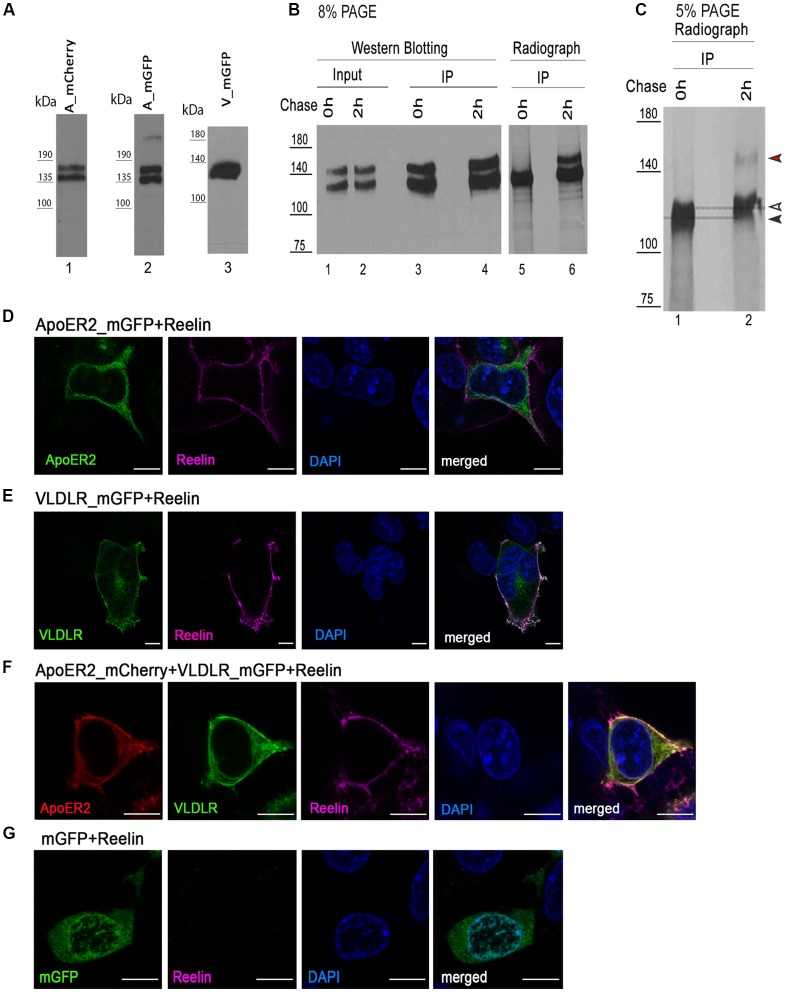
Expression of fluorescently tagged variants of apolipoprotein E receptor 2 (ApoER2) and very low density lipoprotein receptor (VLDLR) in human embryonic kidney cells 293 (HEK293).** (A)** Lysates of HEK293 expressing ApoER2_mCherry (lane 1), ApoER2_mutated/monomeric green fluorescent protein (mGFP; lane 2) or VLDLR_mGFP (lane 3) were analyzed by western blotting using anti-mCherry (lane 1) and anti-GFP (lanes 2–3). **(B)** HEK293 cells expressing ApoER2_human influenza hemagglutinin (HA) were kept for 30 min in starvation medium (w/o methionine and cysteine), then pulse labeled with EasyTag™ EXPRESS^35^S Protein Labeling Mix (200 μCi/ml) for 30 min and chased for the indicated time intervals in DMEM + 10% FCS. Cell lysates were subjected to immunoprecipitation with anti-HA. Cell lysates (input) and precipitates (IP) were separated on 8% **(B)** and 5% **(C)** SDS-PAGE and either analyzed by western blotting using Ab20 (**B**, lanes 1–4) or the gels were dried and analyzed by autoradiography (**B**, lanes 5–6; **C**, lanes 1–2). Black arrow, precursor of ApoER2; white arrow, hypoglycosylated (N-glycosylated) ApoER2; red arrow, hyperglycosylated ApoER2. HEK293 cells expressing ApoER2_mGFP **(D)**, VLDLR_mGFP **(E)**, VLDLR_mGFP and ApoER2_mCherry **(F)**, mGFP **(G)** were incubated with Reelin conditioned medium (RCM) at 37°C for 5 min. After incubation, cells were fixed, stained with DAPI, incubated with mouse anti-Reelin antibody and subsequently with goat anti-mouse IgG DyLight633. Scale bar represents 10 μm.

We expressed VLDLR fused to monomeric GFP (mGFP is a mutated version of GFP which provides the same brightness of fluorescence, does not dimerize, and is more pH-stable than the wild type protein; Zacharias et al., 2002; VLDLR_mGFP) alone or with ApoER2 fused to mCherry (ApoER2_mCherry) in HEK293 cells and tested the expression of the tagged receptors by western blotting and fluorescence microscopy whether they are present at the cell membrane and whether they still bind Reelin. As demonstrated in Figure 1A, ApoER2_mCherry and ApoER_mGFP (see later) are expressed as two distinct variants, both carrying the respective fluorescent tag. This expression pattern reflects exactly the expression pattern of the wt-receptor where always two bands are seen in extracts from total brain and from cells expressing the wt-receptor. These two bands are either precursor and end product as suggested in Li et al. (2001) and Sotelo et al. (2014) or differentially glycosylated variants of the receptor (May et al., 2003; Wasser et al., 2014). To clarify the situation we performed pulse-chase experiments expressing a HA-tagged version of full length ApoER2 in HEK293 cells (Figure 1B, lanes 1–4). The cells were pulse labeled with ^35^S for 30 min and then chased for 2 h with radioactivity-free medium. Immunoprecipitations were performed using an anti-HA antibody at the end of the pulse-period (Figure 1B lanes 3 and 5) and after 2 h of chasing (lanes 4 and 6). The precipitates were analyzed by western blotting using an anti-receptor antibody (lanes 3 and 4) and by autoradiography (lanes 5 and 6). There is only one band present at the end of the pulse-labeling period (lane 5). This band migrates slightly below the lower band of the doublet present in a steady state situation (lane 1) and the lower band present after the chase period (lane 6) suggesting that this is the precursor which gives rise to two differentially glycosylated end products (lane 6). To appreciate this small difference in migration we reevaluated the size difference between the precursor and the faster migrating band of the end products using a 5% PA gel (Figure 1C). On this gel, the precursor and the faster migrating end-product can be separated. In addition, this experiment suggests that the faster migrating product is generated more rapidly than the slower migrating form. This is in agreement with previous data using different glycosidases to evaluate the nature of these variants suggesting that the faster migrating form carries only N-linked sugars and migrates only slightly slower than the non-glycosylated precursor. The variant with the higher rel. molecular mass is the product of both N- and O-glycosylation (Wasser et al., 2014). Analysis of the expression of the tagged receptors by fluorescence microscopy confirms their correct localization at the cell membrane and their ability to bind Reelin (Figures 1D–F). To control for artifacts we expressed soluble mGFP and incubated these cells with Reelin. As seen in Figure 1G, these cells do not bind Reelin.

**Figure 2:**
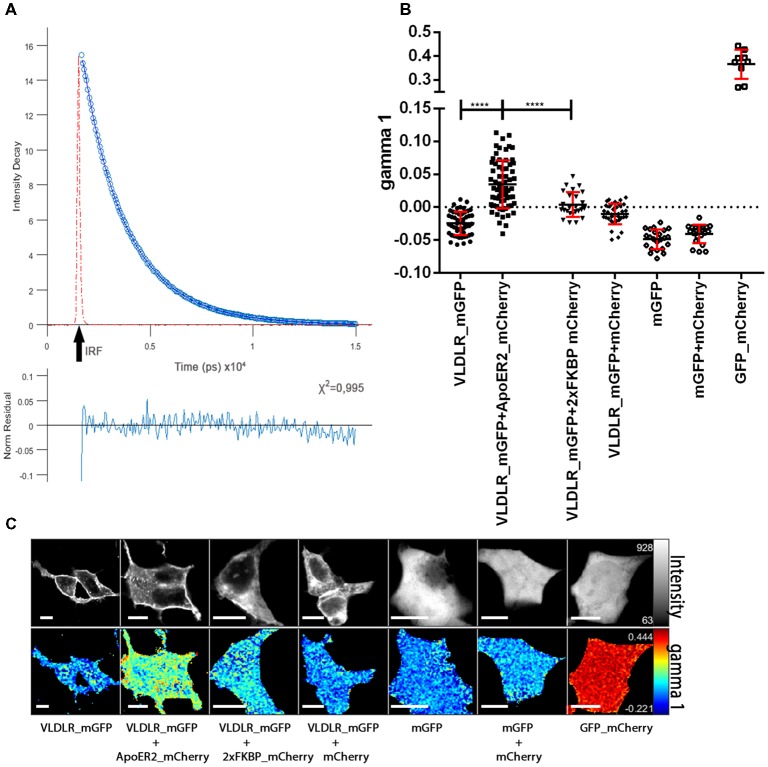
ApoER2 and VLDLR form hetero-oligomers as assessed by fluorescence-lifetime imaging microscopy (FLIM). VLDLR_mGFP was expressed alone or in combination with ApoER2_mCherry or 2xFKBP_mCherry (with membrane localization signal) or soluble mCherry. Soluble mGFP was expressed alone or in combination with soluble mCherry in HEK293 cells. As positive control a fusion protein containing GFP and mCherry was expressed. Interactions of the proteins were analyzed by FLIM Förster resonance energy transfer (FRET). **(A)** Fitted representative fluorescence decay of the donor alone and normalized residues obtained by the chi squared test applied to judge the goodness of the fit for the model used are displayed (IRF, instrument response function). The average of counts from the selected pixels are plotted on the y axis. **(B)** Scatter dot plots illustrating the gamma 1 (FRET contribution with fixed E) of the donor (VLDLR_mGFP) alone and in combination with different acceptors (ApoER2_mCherry, 2xFKBP_mCherry, soluble mCherry) and soluble mGFP alone or together with soluble mCherry, and GFP fused to mCherry from individual cells. Data derived from at least three experiments were analyzed by one-way ANOVA multiple comparisons test; ****p ≤ 0.0001. **(C)** Representative integrated fluorescence intensity and fitted false color gamma 1 maps of VLDLR_mGFP or mGFP in the absence or presence of the indicated acceptors. Scale bars represent 10 μm.

Next, we measured the lifetime of the donor (VLDLR_mGFP) in the absence of the acceptor by pixelwise fitting of the fluorescence images. Figure 2A shows the representative fluorescence decay of the donor in the absence of any acceptor recorded in TCSPC mode. The time resolution of the system is limited and for this reason a model function has to be convoluted based upon the instrument response function (IRF, the shortest lifetime measurable by the instrument). VLDLR_mGFP fluorescence decays were fitted to a bi-exponential decay model. Goodness of the fit for the model used is adequate as judged by *χ*^2^ (chi squared) test which should be around 1 and the residuals should be evenly distributed across the full extent of the data set (Figure 2A, and see “Materials and Methods” section). Live cell FRET imaging, especially time course experiments are usually very challenging because of the necessity of collecting at least thousands of photons and at the same time avoiding photobleaching/toxicity when working with live cells. We decided to analyze our FLIM data using a recently developed FLIMfit software which enables “global fitting applying a multiexponential model under the assumption that the lifetime components are invariant across the image” (Warren et al., 2013, 2015).

For the donor alone (VLDLR_mGFP) the values for the respective lifetimes tau1 and tau2 were 2,522 ns and 1,298 ns and the corresponding values for the lifetime contributions were beta1 = 0.69 and beta2 = 0.31. These values were fixed and used for further data analysis. In order to determine FRET efficiency (E) we used a direct fusion of GFP and mCherry (GFP_mCherry) which should give maximal FRET efficiency (Figures 2B,C). The FRET efficiency for this positive control was 0.5 and was used as fixed value for the analyses of VLDLR_mGFP alone and VLDLR_mGFP in combination with different acceptors. We determined gamma 1 values (contributions of FRET component with fixed E) for every single cell (Figure 2C illustrates fitted false color gamma 1 maps of VLDLR_mGFP in the absence and the presence of different acceptors for representative cells) and the results from all cells are presented in Figure 2B. Gamma 1 values around or below 0 were obtained for the donor alone (VLDLR_mGFP). When VLDLR_mGFP is expressed together with ApoER2_mCherry gamma 1 values significantly increased due to FRET between donor and acceptor. This result suggests that VLDLR and ApoER2 form hetero-di/oligomers even in the absence of Reelin. To test whether this result is due to a specific interaction between both receptors or due to an unspecific effect resulting from high expression levels and overcrowding of the receptors at the plasma membrane of HEK293 cells, we expressed VLDLR_mGFP together with either cytosolic mCherry or mCherry carrying a plasma membrane localization signal and 2xFKBP domains (see “Materials and Methods” section). Proteins carrying one or two FKBP domains can be dimerized or multimerized by the addition of the cell-permeable synthetic ligand AP20187 (Clackson et al., 1998). Co-expression of VLDLR_mGFP together with membrane localized mCherry (2xFKBP_mCherrry) resulted in gamma 1 levels slightly above 0 (Figure 2B). This slight increase is significantly lower in comparison to the increase in gamma 1 in the presence of ApoER2_mCherry. Expression of VLDLR_mGFP together with soluble mCherry also slightly increased gamma 1 which could reflect a different environment around the donor molecule under this condition. Additional controls with soluble GFP alone and together with soluble mCherry demonstrate that there is no significant FRET between the two fluorophores in the cytosol. These controls clearly demonstrate that FRET between VLDLR and ApoER2 is indeed due to a specific interaction and that the two receptors form hetero-oligomers in living cells without addition of any ligand.

**Figure 3:**
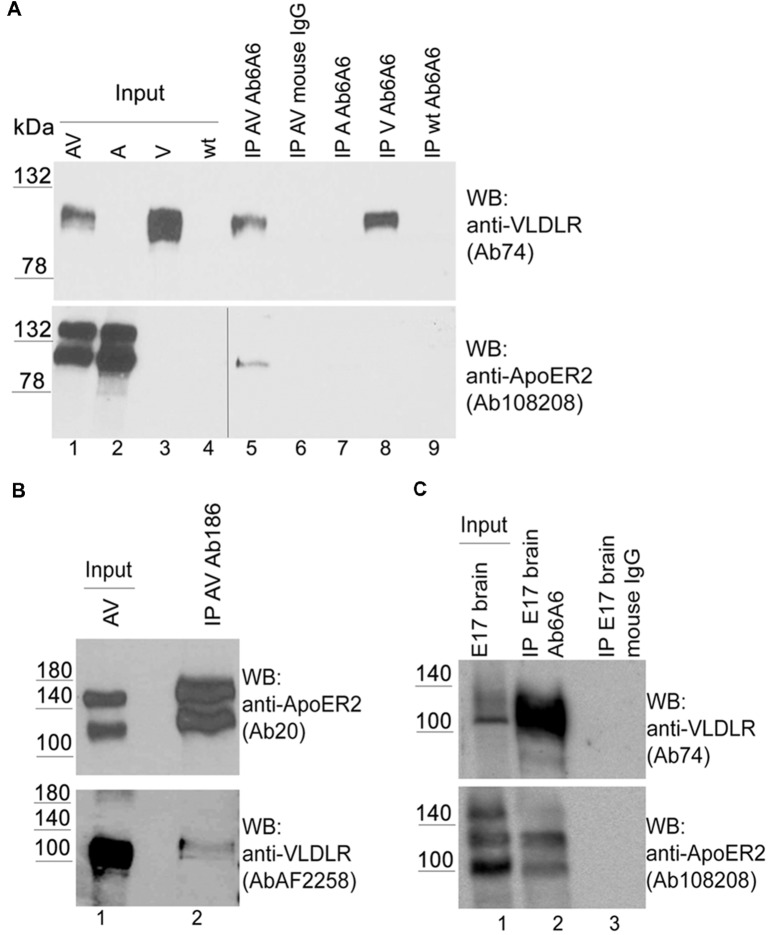
ApoER2 and VLDLR form hetero-oligomers as assessed by immunoprecipitation. HEK293 cells expressing VLDLR and ApoER2 **(A,B)** were lysed and the protein extracts were subjected to immunoprecipitation using **(A)** anti-VLDLR (Ab6A6) and **(B)** anti-ApoER2 (Ab186), or an unrelated antibody (mouse IgG; **A**, lane 6) as control. **(C)** Embryonic rat brain (E17) was lysed and the protein extract was subjected to immunoprecipitation using anti-VLDLR (Ab6A6) or unrelated mouse IgG as control (lane 3). The extracts and precipitates were analyzed by western blotting using the indicated antibodies. Two different exposure times of the same blot are indicated by the vertical line in (**A**; lower part).

This observation prompted us to investigate whether this effect can also be detected by conventional biochemical means. To detect hetero-oligomers we expressed untagged versions of VLDLR or ApoER2 or both receptors together in HEK293 cells (Figure 3A, lanes 1–4) and performed co-immunoprecipitation experiments using precipitating antibodies against VLDLR (Figure 3A) and ApoER2 (Figure 3B), respectively. As demonstrated in Figure 3A (lane 5), precipitation of VLDLR led to the recovery of ApoER2 but preferentially of the hypoglycosylated form. This interaction is specific since an unrelated antibody did not precipitate any of the two receptors (lane 6) and the VLDLR-specific antibody did not precipitate ApoER2 from extracts derived from cells expressing ApoER2 alone (lane 7). When an antibody for ApoER2 was used for precipitation, VLDLR was co-precipitated (Figure 3B). To control for artifacts produced by our cell system used, we performed the same procedure using extracts from embryonic (E17) rat brains. Here, ApoER2 comes in three different variants (Figure 3C, lane 1). The two upper bands are the two differentially glycosylated forms of the receptor (see above); the fastest migrating form with an apparent molecular mass of around 100 kDa is a splice variant lacking exon 16, which codes for the O-linked sugar domain of the receptor. From the mixture of three ApoER2 variants expressed in the embryonic brain (Figure 3C, lane 1) the variant lacking the O-linked sugar domain and the hypoglycosylated but not the hyperglycosylated variant co-precipitates with VLDLR (Figure 3C, lane 2).

### Evaluation of Homo-Oligomerization of Proteins Using TR-FAIM

Next, we evaluated homo-oligomerization of ApoER2 or VLDLR at the basal state. The classical FRET analysis with two different fluorophores (hetero-FRET) can be used to study homo-clustering of the protein of interest. This method, however, is less sensitive since half of the formed homodimers cannot be evaluated (Devauges et al., 2012). Using TR-FAIM circumvents this shortcoming. Here, FRET between the same kinds of fluorophore is measured by loss of anisotropy (i.e., loss of polarization) in the emitted light in comparison to the linearly polarized excitation light.

Loss of anisotropy (loss of polarization) of the emitted light can be caused by rotational movement of a molecule or by energy transfer within the fluorescence lifetime of a fluorophore. To analyze complex time-resolved anisotropy data we applied the FLIMfit software for global fit (Warren et al., 2013, 2015). Global analysis of mGFP or mGFP tagged receptors was performed using a bi-exponential fluorescence decay and bi-exponential anisotropy decay model. Data fitting results in two values of GFP lifetime and two rotational correlation times: θ_1_ (slow component, associated with mobility of the molecule), θ_2_ (very fast component associated with homo-FRET) with respective anisotropy contributions r_1_ (associated with rotational correlation time) and r_2_ (r_FRET_, associated with homo-FRET). We first validated the depolarization due to rotation by using GFP in a solvent of varying viscosity. Global analysis of GFP with varying concentrations of glycerol was performed using a bi-exponential fluorescence decay model and a mono-exponential anisotropy decay model. As expected, decrease in viscosity increased rotation of the fluorophore which led to loss of polarization (lower anisotropy; Supplementary Figures S1A,B) and faster anisotropy decay (Supplementary Figure S1C). Subsequently, depolarization due to FRET was analyzed and calibrated using EGFR as control (Hofman et al., 2010) and ApoER2 and VLDLR fused to FKBP dimerization domain(s) and mGFP which due to a mutation does not form oligomers (Zacharias et al., 2002). The tagged receptors were expressed in HEK293 cells and imaged before and after addition of 1 μM AP20187. As expected rFRET values for monomeric GFP expressed in HEK293 were around 0 which indicates lack of di-/oligomerization of mGFP (Supplementary Figures S2A,C). Forced oligomerization of EGFR_FKBP_mGFP by 1 μM AP20187 produced a significant increase in r_FRET_ (Supplementary Figures S2A,B) as described (Hofman et al., 2010). When ApoER2 and VLDLR fused to one FKBP-domain and mGFP (see “Materials and Methods” section) were expressed, addition of AP20187 significantly increased oligomerization of the receptors (Supplementary Figures S2A,B). Additionally, AP20187 treatment of HEK293 expressing VLDLR fused to a tandem FKBP domain (VLDLR_2xFKBP_mGFP) resulted in even higher r_FRET_ values indicating the formation of higher order oligomers (Supplementary Figure S2A). In comparison to the r_FRET_ values produced by soluble mGFP which is around zero, all of the receptors fused to GFP produced r_FRET_ values significantly above zero. As demonstrated here and for EGFR (Hofman et al., 2010) this is due to the fact that these receptors form already predimers (see below) even in the absence of an oligomerizing ligand or as here in the absence of AP20187.

The algorithm implemented in FLIMfit software allows performing time course experiments with short time intervals. Total integration time per image amounted to about 50 s. Supplementary Figures S3 presents an exemplary time course experiment in which TCSPC images were recorded from cells expressing VLDLR_FKBP_mGFP every few minutes before and after forced receptor oligomerization with 1 μM AP20187. Four minutes after addition of the dimerizer a robust increase in r_FRET_ was recorded indicating clustering of VLDLR_FKBP_mGFP (Supplementary Figures S3A–C).

### ApoER2 and VLDL Receptor Form Homo di/oligomers in the Absence of Reelin

**Figure 4:**
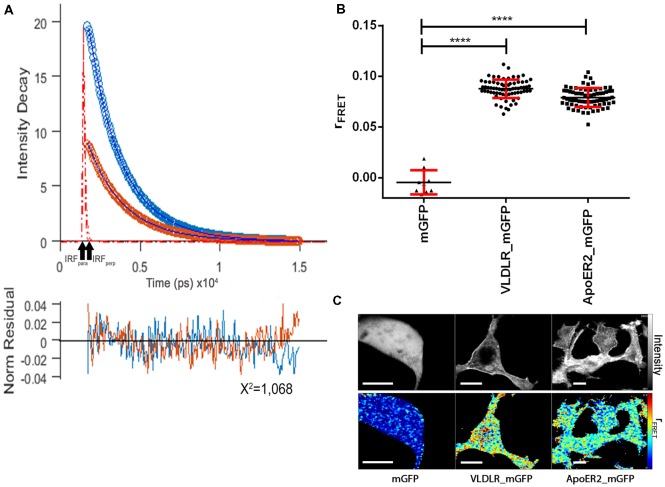
ApoER2 and VLDLR form homo-oligomers as assessed by time-resolved fluorescence anisotropy imaging (TR-FAIM). Soluble mGFP, VLDLR_mGFP, and ApoER2_mGFP were expressed in HEK293 cells. Homo-oligomerization of the receptors was measured by TR-FAIM. Global analysis algorithm was used to fit time-correlated single photon counting (TCSPC) homo-FRET data. **(A)** Fitted representative parallel (blue) and perpendicular (orange) fluorescence decays of VLDLR_mGFP and normalized residues obtained by the chi squared test applied to judge the goodness of the fit for the model used are displayed (IRF, instrument response function). The average of counts from the selected pixels are plotted on the y axis.** (B)** Scatter dot plots illustrate the contribution of anisotropy associated with homo-FRET (rFRET) for soluble mGFP, VLDLR_mGFP and ApoER2_mGFP from individual cells. Data derived from at least three experiments were analyzed by one-way ANOVA multiple comparisons test; ****p ≤ 0.0001. **(C)** Representative integrated fluorescence intensity and fitted false color maps of contribution of anisotropy associated with homo-FRET (rFRET) of soluble mGFP, VLDLR_mGFP, and ApoER2_mGFP. Scale bars represent 10 μm.

Figure 4 presents the respective data sets obtained to evaluate the homo-oligomerization states of VLDLR and ApoER2 fused to monomeric GFP and expressed in HEK293 cells. A representative graph shows the fitted parallel (blue) and perpendicular (orange) fluorescence decays for VLDLR_mGFP (Figure 4A). Fluorescence decays acquired from VLDLR_mGFP and ApoER2_mGFP expressed in HEK293 cells were globally fitted and the values of the fluorescence lifetimes were *τ*_1_ = 2.46 ns and *τ*_2_ = 1.25 ns. The value of the correlation time obtained from anisotropy decays related to rotation was much higher than the one obtained for GFP in the cytosol, demonstrating that the value obtained is indicative for proteins with hindered movement. The value for rFRET for mGFP expressed as cytosolic soluble protein is around 0 (Figure 4B). The corresponding values for VLDLR_mGFP and ApoER2_mGFP, amount to 0.0858 and 0.0777, respectively (Figure 4B). Figure 4C illustrates representative false color maps of rFRET of mGFP, VLDLR_mGFP and ApoER2_mGFP expressed in HEK293. rFRET values for VLDLR_mGFP and ApoER2_mGFP are significantly above 0 which indicates presence of receptor dimers or oligomers without addition of Reelin.

**Figure 5:**
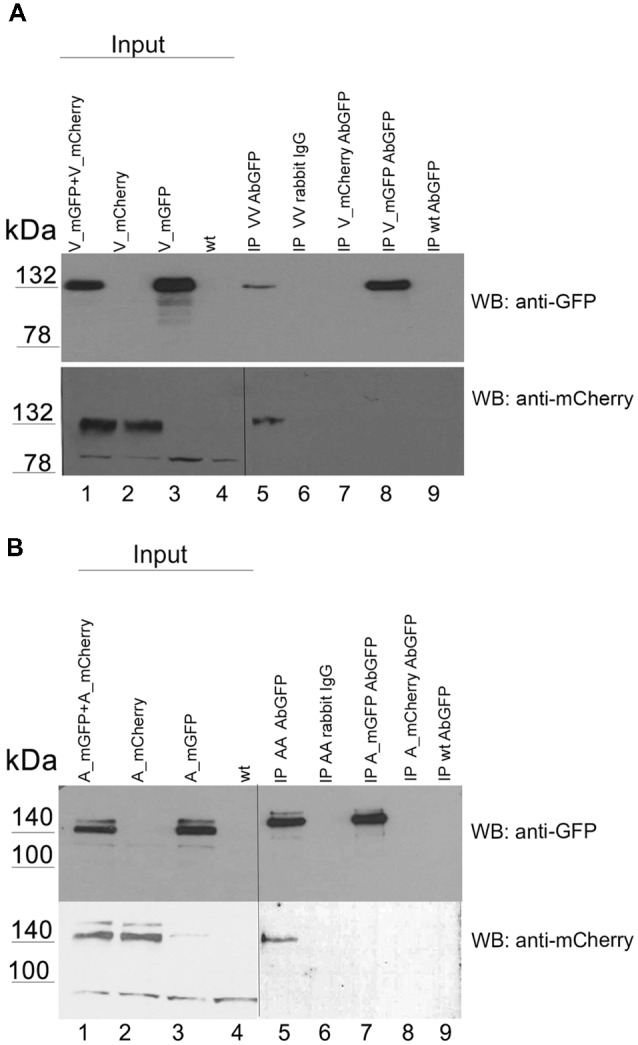
ApoER2 and VLDLR form homo-oligomers as assessed by immunoprecipitation. HEK293 expressing VLDLR_mGFP and VLDLR_mCherry **(A)** or ApoER2_mGFP and ApoER2_mCherry **(B)** were lysed and the protein extracts were subjected to immunoprecipitation using anti-GFP or an unrelated antibody (rabbit IgG; **A,B** lane 6). The precipitates were analyzed by western blotting using the indicated antibodies. Two different exposure times of the same blot are indicated by the vertical line.

To detect receptor homo-oligomers by immunoprecipitation we used tagged variants of the receptors which were also used in our FRET studies. We expressed VLDLR tagged with mGFP or with mCherry together or alone in HEK293 cells (Figure 5A, lanes 1–4) and precipitated the mGFP-tagged receptor from cell extracts using an antibody against GFP (lanes 5, 7–9) or an unrelated control antibody (lane 6). The input and the resulting immunoprecipitates were analyzed by western blotting using an antibody against GFP (upper panel) or against mCherry (lower panel). As demonstrated in Figure 5A (lane 5) precipitation of VLDLR_mGFP co-precipitated the mCherry-tagged version of the receptor. A similar experiment was carried out with two tagged versions of ApoER2 (mGFP, mCherry; Figure 5B). Again, precipitation was performed with an antibody against GFP and the input and the precipitates were tested by western blotting using an antibody against GFP (upper panel) and against mCherry (lower panel). Both tagged versions of ApoER2 are present as hypo- and hyperglycosylated forms, albeit the hyperglycosylated form to a lesser extent (Figure 5B, lanes 1–3). When both receptors are expressed together, immunoprecipitation of GFP co-precipitates the mCherry tagged version of ApoER2 (lane 5), clearly demonstrating that also ApoER2 form di/oligomers in the absence of Reelin.

### Differential Effect of Full Length Reelin and R3–6 on the Increase of Cluster Size of VLDLR/ApoER2 Hetero-Oligomers

**Figure 6:**
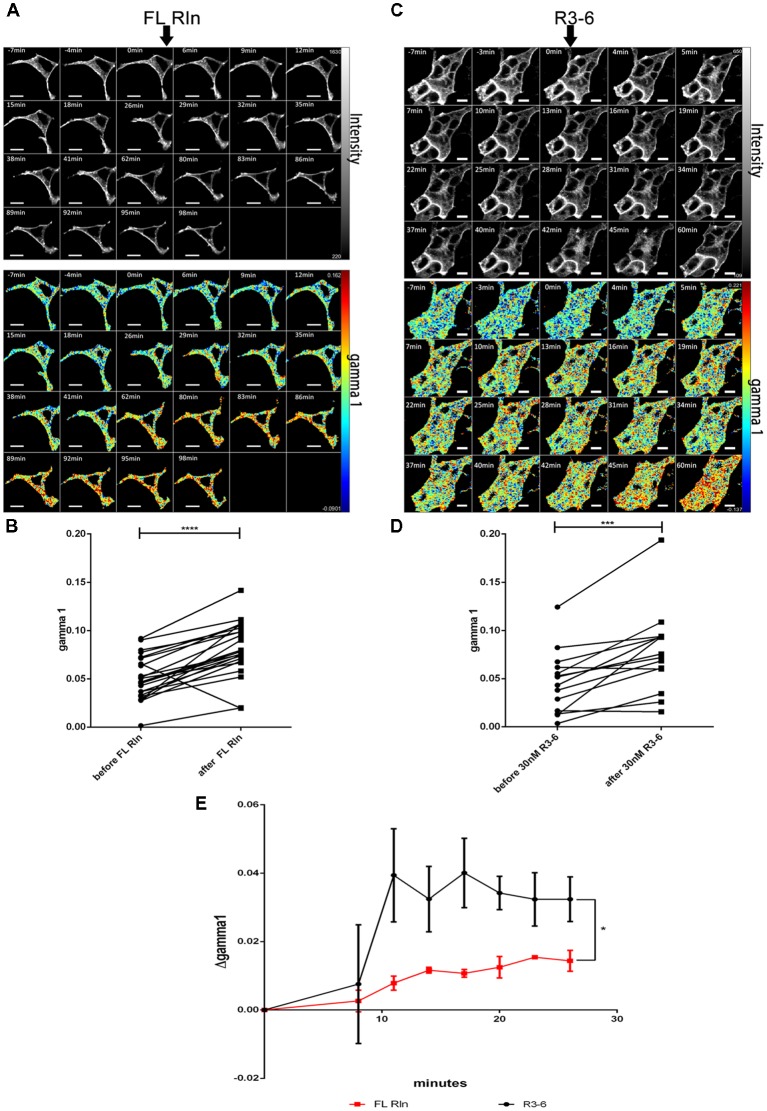
Differential effect of full length Reelin and R3–6 on the increase of cluster size of VLDLR/ApoER2 hetero-oligomers. VLDLR_mGFP and ApoER2_mCherry were expressed in HEK293 cells and interaction of the receptors in the presence of full-length (FL) Reelin **(A,B)** or central fragment of Reelin, R3–6 **(C,D)** was analyzed by FLIM FRET. **(A,C)** Integrated fluorescence intensity images and false color map of gamma 1 over the time course of the experiment. Addition of FL-Reelin or R3–6 is indicated by black arrows. Scale bars represent 10 μm. **(B,D)** Line graphs illustrating gamma 1 values for each cell before and after 1 h of the addition of FL Reelin **(B)** or R3–6 **(D)**. Data derived from 23 cells from three independent experiments **(B)** or from 15 cells from four independent experiments **(D)** were analyzed by paired, two-tailed *t*-test; ***p ≤ 0.001, ****p ≤ 0.0001. **(E)** Time courses (up to 26 min after addition of the ligands) of Δgamma 1 (difference between gamma 1 values before and after addition of the ligand). Black line indicates treatment with FL Reelin and red line with R3–6. Data derived from three independent experiments were analyzed with unpaired Student’s *t*-test. Plots show mean ± SEM; *p ≤ 0.05.

Having established that ApoER2 and VLDLR form hetero-oligomers even in the absence of Reelin, we investigated whether Reelin changes the state of the preformed receptor clusters. Yasui et al. demonstrated that the binding site for ApoER2 and VLDLR is located in the fifth and sixth Reelin repeats (R5–6), mediated by two critical Lys residues (Lys-2360 and Lys-2467; Yasui et al., 2007). In contrast to FL-Reelin, which is always a mixture of different proteolytic fragments, R3–6 can be produced as homogenous recombinant protein (Hirai et al., 2017). Thus, we examined the effect of full length Reelin as well as the central fragment of Reelin (repeats R3–6) on the hetero-oligomerization state of ApoER2 and VLDLR. We started these experiments using RCM which is a mixture of full length (FL) Reelin and a set of defined proteolytic fragments thereof (Jossin et al., 2007). HEK293 cells expressing VLDLR_mGFP (donor) and ApoER2_mCherry (acceptor) were imaged for a few minutes before FL-Reelin was added (Figure 6A). After Reelin addition, gamma 1 increased gradually and reached a maximum after 1 h (Figures 6A,B). R3–6, also increases gamma 1 (Figures 6C,D), however, by analyzing the time course of this effect it became apparent that this increase was significantly different from that of FL-Reelin (Figure 6E). Whereas the effect produced by FL-Reelin was very slow (reaching the maximum only after 60 min), the one induced by the addition of R3–6 became evident already within 5 min and reached robust levels already after 10 min.

### Full Length Reelin but Not R3–6 Increases Cluster Sizes of ApoER2 and VLDLR Homo-Oligomers

**Figure 7:**
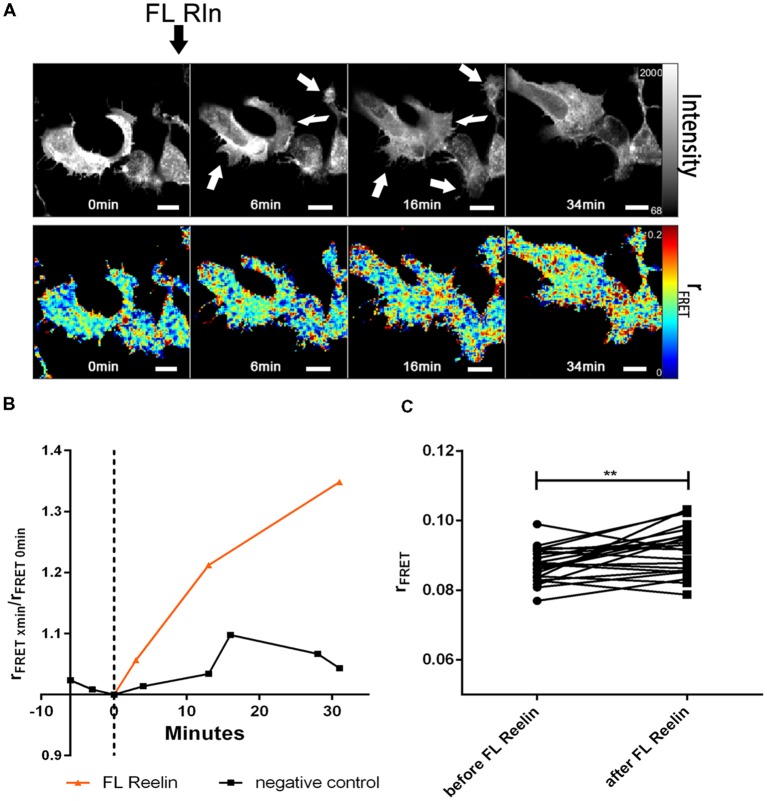
Full length Reelin increases cluster size of ApoER2 homo-oligomers. ApoER2_mGFP was expressed in HEK293 cells. Homo-oligomerization of ApoER2 upon addition of FL Reelin was measured by TR-FAIM. Global analysis algorithm was used to fit TCSPC homo-FRET data. **(A)** Integrated fluorescence intensity images and false color map of contribution of anisotropy associated with homo-FRET (rFRET) over the time course of the experiment. Addition of FL-Reelin is indicated by black arrow. Formation of spikes highlighted with white arrows. **(B)** Representative time course of the anisotropy associated with homo-FRET (expressed in relative numbers based on the value before addition of FL-Reelin; rFRET_0 min_) upon addition of FL-Reelin or negative control. **(C)** Line graph illustrating the contribution of anisotropy associated with homo-FRET (rFRET) before and after treatment for each cell analyzed. Data derived from 22 cells from three independent experiments were analyzed by paired, two-tailed *t*-test; **p ≤ 0.01. Scale bars represent 10 μm.

**Figure 8:**
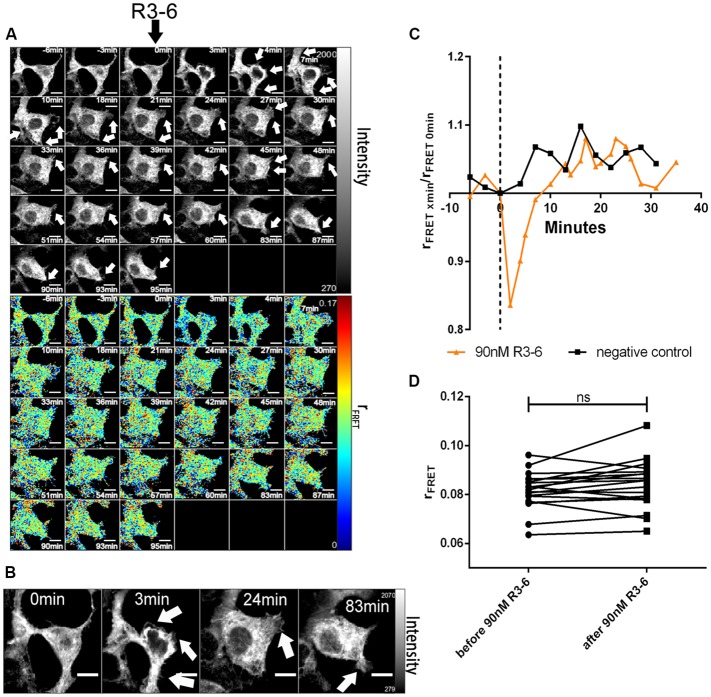
Central fragment of Reelin (R3–6) does not increase cluster size of ApoER2 homo-oligomers. ApoER2_mGFP was expressed in HEK293 cells. Homo-oligomerization of ApoER2 upon addition of R3–6 was measured by TR-FAIM. Global analysis algorithm was used to fit TCSPC homo-FRET data. **(A)** Integrated fluorescence intensity images and false color map of contribution of anisotropy associated with homo-FRET (rFRET) over the time course of the experiment. Addition of R3–6 is indicated by black arrow. Formation of spikes highlighted with white arrows. **(B)** Enlarged images of a cell before and after treatment. Formation of spikes highlighted with white arrows. **(C)** Representative time course of the anisotropy associated with homo-FRET (expressed in relative numbers based on the value before addition of R3–6; rFRET_0 min_) upon addition of R3–6 or negative control. **(D)** Line graph illustrating the contribution of anisotropy associated with homo-FRET (rFRET) before and after treatment for each cell analyzed. Data derived from 19 cells from three independent experiments were analyzed by paired, two-tailed *t*-test; ns, not significant. Scale bars represent 10 μm.

Next, we tested whether FL-Reelin or R3–6 increases cluster sizes of homo-oligomers formed by ApoER2 or VLDLR. TR-FAIM time course experiments with HEK293 expressing only ApoER2_mGFP demonstrated that addition of FL Reelin increased ApoER2 cluster size (Figure 7). In addition, we observed that HEK293 cells expressing ApoER2_mGFP and treated with Reelin changed their shape. Within the first few minutes the cells increased their size and started forming spikes and lamellipodia like structures (highlighted with white arrows in Figure 7A). This effect is reminiscent of the observation that Reelin induces filopodia formation in neurons (Leemhuis et al., 2010). FRET associated anisotropy (r_FRET_) increased 10 min after addition of FL-Reelin, indicating that Reelin increases the size of preformed ApoER2 clusters (Figures 7B,C). Subsequently, we examined the effect of R3–6. Similarly to FL Reelin, R3–6 induced shape changes and multiple spikes started to appear already 3 min after addition of R3–6 (Figures 8A,B, white arrows). Observing the cells for another 90 min revealed that this is a very dynamic process. Already formed spikes disappeared and new ones were rapidly formed during this time period. This is reminiscent to a specific state in neuronal migration during forebrain development where depolarized neuroblasts extend and retract neurites within the MMZ (Tabata and Nakajima, 2003; Tabata et al., 2009). Whether the same effect seen with FL-Reelin (see above) is indeed produced by FL-Reelin or by R3–6, which is always present in Reelin preparations remains to be established.

In sharp contrast to FL-Reelin, however, even at the highest concentration used (90 nM), R3–6 did not significantly change r_FRET_ indicating that the central fragment alone does not increase ApoER2 cluster size (Figures 8C,D). The obvious and reversible decrease in r_FRET_ just after stimulation (Figure 8C) is caused by temperature changes after opening the chamber in which cells were cultivated. The same effect was also sometimes observed in negative controls.

**Figure 9:**
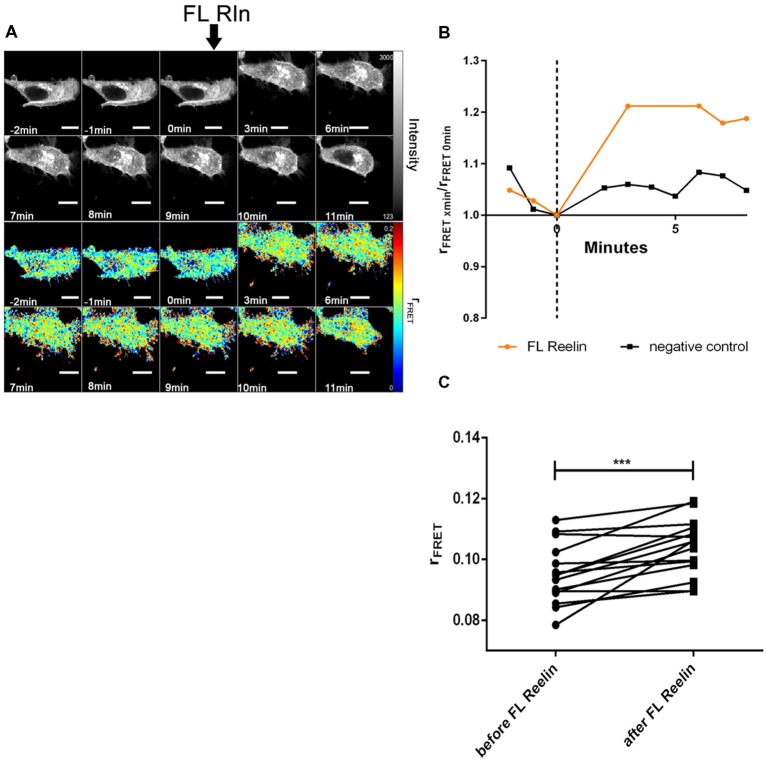
Full length Reelin increases cluster size of VLDLR homo-oligomers. VLDLR_mGFP was expressed in HEK293 cells. Homo-oligomerization of VLDLR upon addition of FL-Reelin was measured by TR-FAIM. Global analysis algorithm was used to fit TCSPC homo-FRET data. **(A)** Integrated fluorescence intensity images and false color map of contribution of anisotropy associated with homo-FRET (rFRET) over the time course of the experiment. Addition of FL-Reelin is indicated by black arrow. **(B)** Representative time course of the anisotropy associated with homo-FRET (expressed in relative numbers based on the value before addition of FL-Reelin; rFRET_0 min_) upon addition of FL-Reelin or negative control. **(C)** Line graph illustrating the contribution of anisotropy associated with homo-FRET (rFRET) before and after treatment for each cell analyzed. Data derived from 15 cells from three independent experiments were analyzed by paired, two-tailed *t*-test; ***p ≤ 0.001. Scale bars represent 10 μm.

**Figure 10:**
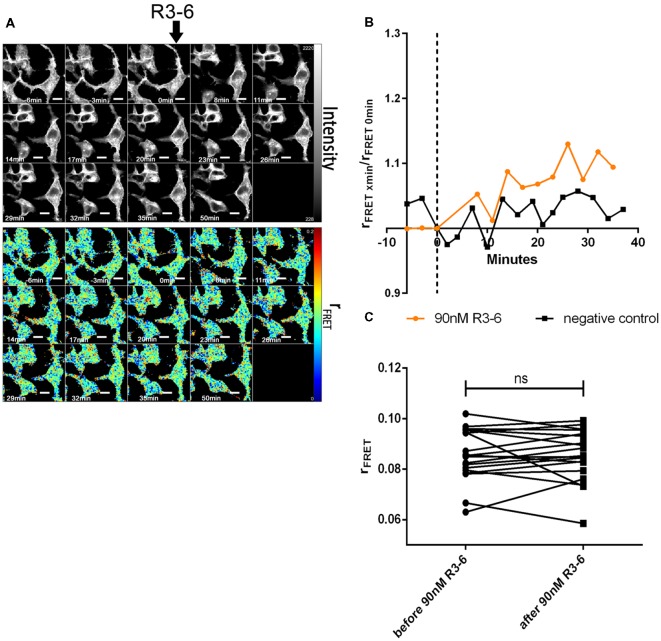
Central fragment of Reelin (R3–6) does not increase cluster size of VLDLR homo-oligomers. VLDLR_mGFP was expressed in HEK293 cells. Homo-oligomerization of VLDLR upon addition of R3–6 was measured by TR-FAIM. Global analysis algorithm was used to fit TCSPC homo-FRET data. **(A)** Integrated fluorescence intensity images and false color map of contribution of anisotropy associated with homo-FRET (rFRET) over the time course of the experiment. Addition of R3–6 is indicated by black arrow. **(B)** Representative time course of the anisotropy associated with homo-FRET (expressed in relative numbers based on the value before addition of R3–6; rFRET_0 min_) upon addition of R3–6 or negative control. **(C)** Line graph illustrating the contribution of anisotropy associated with homo-FRET (rFRET) before and after treatment for each cell analyzed. Data derived from 19 cells from two independent experiments were analyzed by paired, two-tailed *t*-test; ns, not significant. Scale bars represent 10 μm.

Finally, we evaluated the effect of FL-Reelin and R3–6 on VLDLR homo-oligomerization and obtained the same results as for ApoER2. FL-Reelin induces rapid increase of oligomerization of VLDLR (Figure 9). R3–6 even at a concentration of 90 nM does not change the oligomerization state of VLDLR (Figure 10). In contrast to cells expressing ApoER2, VLDLR expressing cells did not form filopodia upon treatment with R3–6, but diminished in size (see below).

To quantify the effect of R3–6 on cells expressing ApoER2, VLDLR, or both in respect to the observed dynamic changes in cell shape we analyzed the observed dynamic changes in cell morphology using ImageJ (1.52i., USA). We applied the particle measurement feature to measure cell-area, cell-perimeter, cell-solidity, and Feret diameter. As demonstrated for three independent cells expressing ApoER2 over a time period of 40 min (Figures 11.1A–D; red lines), shape parameters changed over a wide range when these cells were treated with R3–6. In these cells the cell-area significantly increased. For control cells, kept in imaging medium, these parameters only minimally changed during the same time period (black lines). Cells expressing VLDLR (Figures 11.2A–D, red lines) significantly decreased in cell size, a process which is also accompanied by a highly dynamic change in the other shape parameters measured (cell-perimeter, cell-solidity, and Feret diameter). Both specific effects (increase in cell size induced by ApoER2 and cell shrinkage by VLDLR) are abolished when both receptors are present at the same time (Figures 11.3A–D).

**Figure 11:**
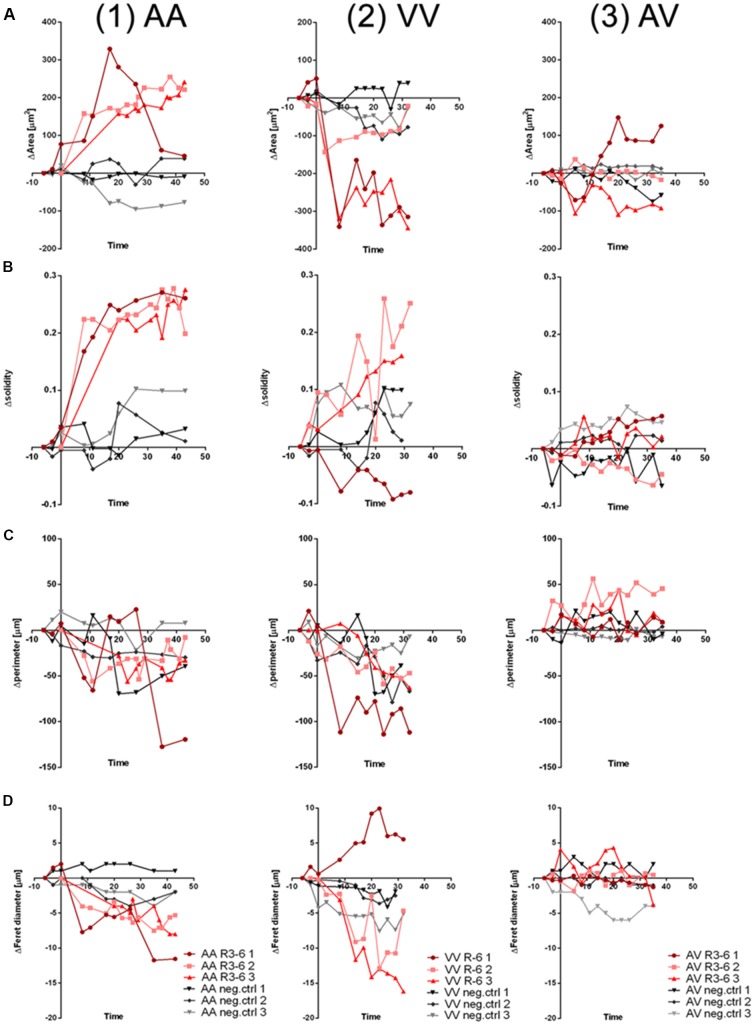
Cell morphology changes upon R3–6 interaction with ApoER2 and VLDLR. Changes in cell morphology parameters (**A**, ΔArea;** B**, Δsolidity; **C**, Δperimeter; **D**, ΔFeret diameter) of HEK293 cells expressing ApoER2_mGFP (AA, column 1); VLDLR_mGFP (VV, column 2) or ApoER2_mCherry and VLDLR_mGFP (AV, column 3) which were treated either with R3–6 (red curves) or imaging medium (negative control, gray curves) are presented for three time courses for each condition.

**Figure 12:**
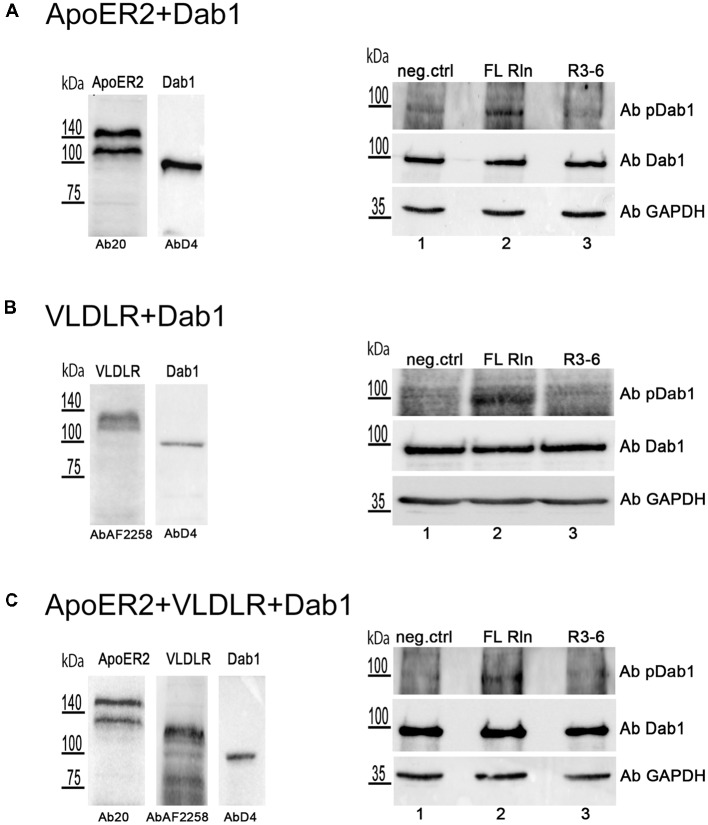
Central fragment of Reelin (R3–6) does not induce Dab1 phosphorylation. HEK293 cells expressing Dab1_myc and ApoER2 **(A)** or Dab1_myc and VLDLR **(B)** or Dab1_myc, ApoER2, and VLDLR **(C)** were kept in Imaging Medium for 30 min. Next, the medium was changed to RCM containing full length Reelin (FL Rln, lanes 2) or R3–6 (lanes 3) was added to the cells (final concentration, 30 nM) or cells were left untreated (neg.ctrl, lanes 1). After 20 min incubation at 37°C, cells were washed and lysed in NP-40 lysis buffer. Lysates were analyzed by western blotting using phospho-Dab1 antibody (Ab pDab1) and GAPDH antibody. Membranes were stripped and subsequently re-probed for the detection of Dab1 (AbD4). ApoER2 was detected by Ab20 and VLDLR by AbAF2258.

These observations prompted us to test whether R3–6 would be able to induce Dab1 phosphorylation in the cell system used for this study. HEK293 cells were transiently transfected with myc-tagged Dab1 in combination with either ApoER2 (Figure 12A) or VLDLR (Figure 12B) or with both receptors together (Figure 12C). These cells were treated with either FL-Reelin (lanes 2) or R3–6 (lanes 3), or mock control (lanes 1) and phosphorylation of Dab1 was evaluated by western blotting using a phospho-Dab1 specific antibody. The amount of total Dab1 was assessed using a Dab1-specific antibody (D4). In contrast to FL-Reelin, which induces a robust phosphorylation of Dab1 *via* ApoER2 or VLDLR (Figures 12A,B; lanes 2), R3–6 failed to produce this effect (lanes 3). Interestingly, R3–6 also failed to induce a robust Dab1 phosphorylation, when both receptors are present (Figure 12C, lane 3). These results suggest that Dab1 phosphorylation is mainly mediated by increasing cluster sizes of homo-oligomers of ApoER2 or VLDLR by FL-Reelin. Interactions of R3–6 with ApoER2 or VLDR leads to cell shape rearrangements and might be part of a signaling pathway different from the canonical Reelin pathway.

## Discussion

Development of the mammalian neocortex is a very complex process in that the Reelin signaling pathway plays a pivotal role. The well-established core of this pathway involves, in a consecutive order, binding of Reelin to its receptors ApoER2 and/or VLDLR and subsequent phosphorylation of Dab1. How this simple pathway can induce signals leading to many different cellular actions such as orientation of migration of multipolar neuroblasts, induction of branching, stop of migration, and detachment from glial fibers is hard to perceive. One possible option to untangle this problem is to analyze the different variants of the proteins involved in this pathway in terms of their spatiotemporal expression and their specific molecular action. Such an approach has recently led to the discovery that alternative splicing of Dab1 controls multipolar to bipolar transition of migrating neurons in the neocortex (Zhang et al., 2018). Another heterogeneous player is Reelin itself. It is expressed in the marginal zone (Hirota et al., 2015) by Cajal-Retzius cells (Ogawa et al., 1995) but also by some cells in the subventricular zone (SVZ) and intermediate zone (Uchida et al., 2009) where it promotes neurogenesis in cooperation with Notch (Lakomá et al., 2011). In addition, specific proteolysis of FL-Reelin produces a set of defined proteolytic fragments (Jossin et al., 2007), which possibly differ in their function and distance to diffuse. But the most complex situation is created by the Reelin receptors. First, two different receptors namely ApoER2 and VLDLR exist (Trommsdorff et al., 1999) which have different affinities for Reelin (Andersen et al., 2003) and are expressed in different regions of the developing neocortex (Hirota et al., 2015). In our study we have focused on the receptors and used time-resolved anisotropy and FLIM to study the specific interactions between the receptors upon binding of Reelin and its central fragment.

The major results of these efforts are: (1) in the absence of Reelin the receptors already form clusters which can contain either of the receptors alone or a combination of both receptors; (2) FL Reelin but not R3–6 increases the size of ApoER2 and VLDLR homo-clusters. ApoER2/VLDLR hetero-clusters are only weakly increased by FL-Reelin; (3) the central Reelin fragment (R3–6) does not induce Dab1 phosphorylation by any combination of the receptors; and (4) R3–6 induces a dynamic process of spike/lamellipodia formation and increase in cell size when ApoER2 is the only receptor present and a reduction in cell size when acting on cells expressing VLDLR only.

What is the basic event which leads to Dab1 phosphorylation? The current concept is that Reelin induces clustering of Dab1 *via* oligomerization of the receptors which are monomers prior to ligand binding. Here, we show that the receptors form dimers/oligomers already at the basal state i.e., in the absence of Reelin as demonstrated by very sensitive FLIM/TR-FAIM analyses. This was already suggested before by biochemical means (Divekar et al., 2014). In this publication, basal clustering of ApoER2 in the absence of any ligand was demonstrated. In addition, it was shown that clustering of ApoER2 increases upon Reelin treatment. Based upon these finding together with our own precipitation experiments which gave the same results, we are very confident that the methods used here (time-resolved anisotropy and FLIM) are well suited to study this effect in close detail. Our findings are reminiscent of recent discoveries about EGF-receptor (Hofman et al., 2010) and in general for other receptor tyrosine kinases (Maruyama, 2014) which were originally thought to function as monomers being dimerized or oligomerized by their ligands. These receptors are also present as pre-formed, yet inactive, dimers before being activated by their ligands which induce higher order clusters of the receptors upon binding. Thus, we have to assume that the canonical Reelin signal is induced by increasing the cluster size of the receptors. The receptor binding site of Reelin is located within R5–6 (Yasui et al., 2007) and the cysteine important for forming Reelin homodimers is located in R5 (Yasui et al., 2011) defining the central fragment of Reelin R3–6 as the minimal functional unit of biologically active Reelin. As demonstrated here, this fragment is not sufficient to increase the size of ApoER2 or VLDLR homo-clusters and does not induce Dab1 phosphorylation in HEK293 cells, even when they express both receptors. This is surprising since in contrast to FL-Reelin, R3–6 increases the cluster size of ApoER2/VLDLR hetero-oligomers. At the moment we have no explanation for this behavior. In addition, it has to be pointed out that the result that R3–6 does not induce Dab1 phosphorylation in HEK293 cells in the presence of any combination of ApoER2 and VLDLR is opposite to previously published data, which show that the central Reelin fragment can induce Dab1 phosphorylation in postmitotic neurons (Jossin et al., 2004; Yasui et al., 2007; Lee et al., 2014). This could mean that the mechanism by which R3–6 induces Dab1 phosphorylation in postmitotic neurons is different from that in the HEK293 model used here. Our results, however, are in line with the fact that the monoclonal antibody CR-50, which recognizes an epitope in the N-terminal region of Reelin, disrupts its function (Nakajima et al., 1997). In addition, it was shown that: (i) mutated Reelin, which lacks the CR-50 epitope region, cannot form homopolymers and fails to induce Dab1 phosphorylation in postmitotic neurons (Utsunomiya-Tate et al., 2000; Kubo et al., 2002); and (ii) homo-dimerization of Reelin is important for its function suggesting that an intact higher order architecture of FL-Reelin multimers is essential to increase cluster size of the receptor oligomers and thus for its full biological activity (Yasui et al., 2011). This view is also in line with recent results demonstrating that the secreted metalloproteinase A disintegrin and metalloproteinase with thrombospondin motifs 3 (ADAMTS-3) which is responsible for proteolytic cleavage of the N-terminal fragment of Reelin inactivates Reelin *in vitro* (Koie et al., 2014) and *in vivo* (Ogino et al., 2017).

**Figure 13:**
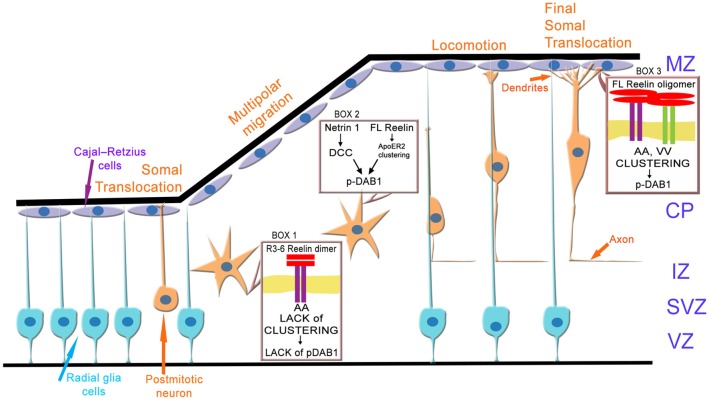
Model of ApoER2 and VLDLR mediated signaling during cortical development. Radial glia cells divide asymmetrically to produce postmitotic neurons. During early stages of cortical development these neurons migrate *via* somal translocation. When the thickness of the cerebral wall increases neurons switch their migration mode to multipolar migration followed by locomotion. Thus, polarized neurons leave the ventricular (VZ) and subventricular zone (SVZ), depolarize and migrate through the IZ *via* a multipolar migration mode. The depolarization step is ill defined and may involve ApoER2 (A)-mediated signaling driven by the central Reelin fragment R3–6 (box 1). Repolarization of the neurons as prerequisite to glia cell guided locomotion is induced by Dab1 phosphorylation mediated by a netrin 1—deleted in colon cancer (DCC) signaling cascade and/or by the canonical Reelin pathway (box 2). Locomotion and terminal translocation is orchestrated by a complex set of Reelin (full length, FL) signals transmitted by clustering of ApoER2 (A) and VLDLR (V) homo- oligomers, leading to Dab1 phosphorylation (box 3). CP, cortical plate; IZ, intermediate zone; MZ, marginal zone.

How can these results now be integrated into the current model (Figure 13) of neuronal migration and layer formation (Sekine et al., 2014; Bock and May, 2016; Chai and Frotscher, 2016; Kon et al., 2017; Santana and Marzolo, 2017)? Initially, newborn neurons change from a bipolar to a multipolar shape when they enter the intermediate or MMZ. In this zone, neuroblasts express ApoER2 but no VLDLR (Uchida et al., 2009; Hirota et al., 2015). Since FL Reelin is not able to diffuse within the cortical plate (Jossin et al., 2007), predominantly the central fragment (R3–6) derived from FL-Reelin produced in the MZ might be present in the MMZ where its function is critical for the development of the cortical plate (Jossin et al., 2004). The results presented here demonstrate that the signal produced by binding of R3–6 to ApoER2 in this zone may not increase the size of ApoER2 clusters and Dab1 seems not to be phosphorylated by this process. However, FL-Reelin is expected to be also present in the SVZ and the lower part of the intermediate zone (Uchida et al., 2009). FL-Reelin in these areas is expected to induce Dab1 phosphorylation by clustering ApoER2 homo-oligomers (Figure 13, box 2). The importance of Dab1 phosphorylation in this zone, however, is still not completely resolved. Previous results have suggested that Dab1 phosphorylation in the MMZ is important since inhibition of Dab1 degradation, which is triggered by its phosphorylation, perturbs CP development (Bock et al., 2004; Feng et al., 2007). Conditional knock out of Dab1 on the other hand, demonstrated that Dab1 is not required for initial polarization or process extension of neurons within in the MMZ (Franco et al., 2011). In addition, rescue experiments using embryonic brain slice cultures demonstrated that Dab1 phosphorylation alone is not sufficient to transmit the entire Reelin signal (Jossin et al., 2004). As demonstrated here, the signal generated by R3–6 *via* ApoER2 induces the formation of spikes and lamellipodia and might induce depolarization of the postmitotic neurons (Figure 12, box1). Re-polarization of these cells which depends on the stimulation of LIMK1 and cofilin and stabilization of the leading process (Chai et al., 2016) might be different from the canonical Reelin pathway. In addition, it was recently demonstrated that deleted in colorectal cancer (DCC) binds netrin1 and this interaction leads to Dab1 phosphorylation (Figure 12, box2; Zhang et al., 2018). Knock-down of DCC impairs multipolar-to-bipolar transition and interrupts proper migration of neuroblasts through this zone. Within the cortical plate close to the marginal zone where Reelin is secreted, the predominant Reelin receptor is VLDLR which is expressed on the leading processes of migrating neurons (Hirota et al., 2015). ApoER2 is also expressed to a lesser extent and mostly on different cellular structures or cells than VLDLR. Thus, in this zone of the developing cortex all possible players of the canonical Reelin pathway are present: full length Reelin, R3–6, VLDLR, ApoER2, and Dab1. Cells expressing VLDLR only will present VLDLR homo-oligomers on their surface. Whether the observed retraction of cell size induced by R3–6 in cells expressing VLDLR only plays a role in stopping migrating neurons in this zone remains to be established. Cells expressing ApoER2 only will present preformed ApoER2 clusters on their surface. According to our results FL Reelin will increase the cluster size of these receptors and will induce Dab1 phosphorylation (Figure 13, box3). The small population of cells expressing both receptors (Hirota et al., 2015) at the same time are expected to have ApoER2 homo-oligomers in their rafts and VLDLR homo-oligomers and ApoER2/VLDLR hetero-oligomers in the non-raft fraction of the cell membrane. As demonstrated here and suggested by others (May et al., 2003; Wasser et al., 2014) the two forms of ApoER2 are differentially glycosylated variants of the receptor. The larger form resides exclusively in rafts whereas the hypoglycosylated variant is found together with VLDLR in the non-raft fraction of the cell membrane (Mayer et al., 2006; Duit et al., 2010). This is supported by our results from co-immunoprecipitation studies (Figure 3) where it is demonstrated that in the basal state only the hypoglycosylated form of ApoER2 precipitated with VLDLR. According to the results presented here, ApoER2/VLDLR hetero-oligomers which are present in the non-raft fraction may not contribute to the levels of phosphorylated Dab1. In addition, R3–6 in combination with ApoER2 or VLDLR on selected subsets of neurons present in this zone might produce signals different from the canonical Reelin signal. Such a splitting of signals might explain suggested differential (Hack et al., 2007) and/or similar (Hirota et al., 2018) roles of ApoER2 and VLDLR in the marginal zone.

Finally, we would like to turn to the effect that R3–6 induces changes in cell shape in HEK293 cells depending on the type of receptor expressed. It remains to be established whether FL-Reelin itself or small amounts of R3–6, which are always present in a preparation containing FL-Reelin, can induce this effect. When R3–6 was applied to cells expressing ApoER2 formation of filopodia was induced, the size of the cells significantly increased and cell shape parameters (cell-perimeter, cell-solidity, and Feret diameter) start to fluctuate. When VLDLR is the only receptor present, R3–6 also induces significant fluctuations in these parameters although the results of this process is different. The cells do not form filopodia but shrink and consolidate their size. Why these effects are lost when both receptors are present at the same time is not clear yet. It has to be pointed out, however, that only a small number of cells in the marginal zone express both receptors (Hirota et al., 2015). The mechanism leading to these effects seems to be different from the canonical Reelin pathway since R3–6 does not induce Dab1 phosphorylation in HEK293 cells. Whether this process is dependent on Dab1 cannot be excluded at this moment. To this end we could demonstrate that HEK293 cells express Dab1 (Supplementary Figure S4A, lane 1, and Supplementary Figure S4B, lane 2). It was shown that Dab1 expressed in COS7 cells associates with Neural Wiskott-Aldrich syndrome protein (N-WASP) and induces actin polymerization through the actin-related protein 2/3 (Arp2/3) complex (Suetsugu et al., 2004) leading to filopodia formation. This process, however, is blocked by Dab1 phosphorylation. This would be in agreement with data presented here, that R3–6 present in the MMZ induces filopodia formation without increasing cluster size of ApoER2 homo-oligomers. Since very little is known about how newly generated neuroblasts acquire a multipolar morphology when they exit the ventricular zone this serendipitous finding might trigger further studies to tackle this problem.

We have to point out, that all experiments presented here have been performed in HEK293 cells and it is not clear at this point whether all conclusions drawn can be correlated with the *in vivo* situation. However, these cells share features with neurons, and might well be suited to study general processes related to the Reelin pathway (Shaw et al., 2002). The main findings that ApoER2 and VLDLR form receptor homo- and hetero-oligomers and that the canonical Reelin signal is generated by increasing the cluster sizes of the receptors are expected to hold true to be the molecular mechanism for the activation of this pathway. Whether the suggested function of the central Reelin fragment (R3–6), which seems to be independent from Dab1 phosphorylation exists *in vivo* and how it blends into the complex processes orchestrating the architecture of the cerebral cortex remains to be established.

## Author Contributions

JN and PD contributed to conception and design of the study and wrote the manuscript. PD performed pulse chase, co-immunoprecipitation and FRET experiments. RT performed immunofluorescence and expression studies. PD prepared the figures under supervision of JN. JN provided guidance and supervision.

## Conflict of Interest Statement

The authors declare that the research was conducted in the absence of any commercial or financial relationships that could be construed as a potential conflict of interest.

## Abbreviations

ADAMTS-3: A disintegrin and metalloproteinase with thrombospondin motifs 3
ApoER2: apolipoprotein E receptor 2
Arp2/3: actin-related protein 2/3
COS-7: immortalized CV-1 cells from the kidney of the African green monkey
Dab1: disabled-1
DCC: Deleted in Colorectal Carcinoma
EGFR: epidermal growth factor receptor
FKBP: FK506 binding protein
FL: full length
FLIM: fluorescence-lifetime imaging microscopy
FRET: Förster resonance energy transfer
HA: human influenza hemagglutinin
HEK293: human embryonic kidney cells 293
IRF: instrument response function
LDL: low-density lipoprotein
mGFP: mutated/monomeric green fluorescent protein
NA: numerical aperture
NIH 3T3: mouse embryonic fibroblast cell line
N-WASP: Neural Wiskott-Aldrich syndrome protein
PI3K: phosphatidylinositide 3-kinase
R3–6: Reelin central fragment, repeats R3-R6
Rap1: Ras-proximate-1
RCM: Reelin-conditioned medium
SVZ: subventricular zone
TCSPC: Time-Correlated Single Photon Counting
TR-FAIM: time-resolved fluorescence anisotropy imaging
VLDLR: very low density lipoprotein receptor.
