# Supplementary material for: Differential Action of Reelin on Oligomerization of ApoER2 and VLDL Receptor in HEK293 Cells Assessed by Time-Resolved Anisotropy and Fluorescence Lifetime Imaging Microscopy
Source: Front Mol Neurosci. 2019 Feb 26;12:53. doi: 10.3389/fnmol.2019.00053 (PMC6403468; doi:10.3389/fnmol.2019.00053)
Supplement: Supplementary file 1 [file Table_1.docx]

**Differential action of Reelin on ApoER2 and VLDL receptor assessed by time-resolved anisotropy and fluorescence lifetime imaging microscopy**

Paula Dlugosz, Roland Tresky, Johannes Nimpf^1^

^1^Department of Medical Biochemistry, Max F. Perutz Laboratories, Medical University Vienna, 1030 Vienna, AUSTRIA

**Supporting Information:**

**Figure S1. Depolarization of GFP due to rotation in dependence of the viscosity of the solvent**

**Figure S2. Depolarization due to FRET by forced oligomerization of receptor molecules**

**Figure S3. AP20187 induces VLDLR_FKBP_mGFP dimerization**

**Figure S4. Dab1 expression in HEK293**

**Legends to Figures (supporting information)**

**Figure S1. Depolarization of GFP due to rotation in dependence of the viscosity of the solvent**

**(A)** Representative images from each condition showing false color map of r_1_. **(B)** Values obtained from global fit of GFP in solvents of varying viscosity. Tau_1_,tau_2_, lifetimes of the fluorophore; beta_1_, beta_2_, fractional contributions of each lifetime; theta_1, the rotational correlation time associated with mobility of the molecule; r_1,_ anisotropy contribution associated with the rotational correlation time; r_ss_, steady-state anisotropy. Data were derived from 3 experiments. Scale bars represent 10µm. **(C**) The anisotropy decays of GFP in solvents of varying viscosity. Black line, decay of 5µM of GFP in 50% glycerol; red line, decay of 5µM of GFP in 25% glycerol.

**Figure S2. Depolarization due to FRET by forced oligomerization of receptor molecules**

**(A)** Scatter dot plots illustrating the contribution of anisotropy associated with homo-FRET (rFRET) before and after forced oligomerization of receptors fused to mGFP and one FKBP (one dimerization domain) or 2xFKBP (two dimerization domains) upon treatment with AP20187. Values obtained from global fit of mGFP alone **(C)** or receptors tagged with FKBP and mGFP **(B)** expressed in HEK293 cells. Tau_1_,tau_2_, lifetimes of the fluorophore; beta_1_, beta_2_, fractional contributions of each lifetime; theta_1, the rotational correlation time associated with mobility of the molecule; theta_2, the rotational correlation time associated with homo-FRET; r_2 (_r_FRET),_ anisotropy contribution associated with homo-FRET. Data were analyzed by two-tailed t-test; *p ≤0.05, **p ≤0.01, ****p ≤ 0.0001.

**Figure S3. AP20187 induces VLDLR_FKBP_mGFP dimerization**

VLDLR_FKBP_mGFP was expressed in HEK293 cells and anisotropy associated with homo-FRET was measured. **(A)** Integrated fluorescence intensity images and false color map of contribution of anisotropy associated with homo-FRET (rFRET) over the time course of the experiment. Addition of 1µM AP20187 is indicated by black arrow. **(B)** Representative time course of the anisotropy associated with homo-FRET (rFRET) upon addition of AP20187 (0min). **(C)** Line graph illustrating the contribution of anisotropy associated with homo-FRET (rFRET) before and after addition of AP20187 for each cell analyzed. Data derived from 12 cells from 2 independent experiments were analyzed by paired, two-tailed t-test, ****p ≤ 0.0001. Scale bars represent 10µm.

**Figure S4. Dab1 expression in HEK293**

**A)** RNA from HEK293 was extracted and reverse transcribed using the RevertAid H Minus First Strand cDNA Synthesis Kit (Thermo Scientific) using oligo (dT)_18_ primers. Subsequently, PCR using primers specific for complete cDNA sequence of human Dab1 was performed and the band with the expected size of about 1700bp corresponding to human Dab1 (lane 1) was detected by agarose gel electrophoresis. As positive control a plasmid carrying the Dab1 sequence was used. Negative controls used in this study: RT-, no reverse transcriptase; NTC, no RNA template; PCR neg, no cDNA template. **B)** Protein lysates from HEK293 cells (lane 1) and NIH3T3 VD cells (stably expressing murine VLDLR lacking the O-linked sugar domain and murine Dab1, lane 2) were analyzed by western blotting using a specific anti-Dab1 antibody (AbD4). Vertical line indicates that bands presented here are derived from different parts of the same gel.
