# Supplementary figures and images for: Differential Action of Reelin on Oligomerization of ApoER2 and VLDL Receptor in HEK293 Cells Assessed by Time-Resolved Anisotropy and Fluorescence Lifetime Imaging Microscopy
Source: Front Mol Neurosci. 2019 Feb 26;12:53. doi: 10.3389/fnmol.2019.00053 (PMC6403468; doi:10.3389/fnmol.2019.00053)

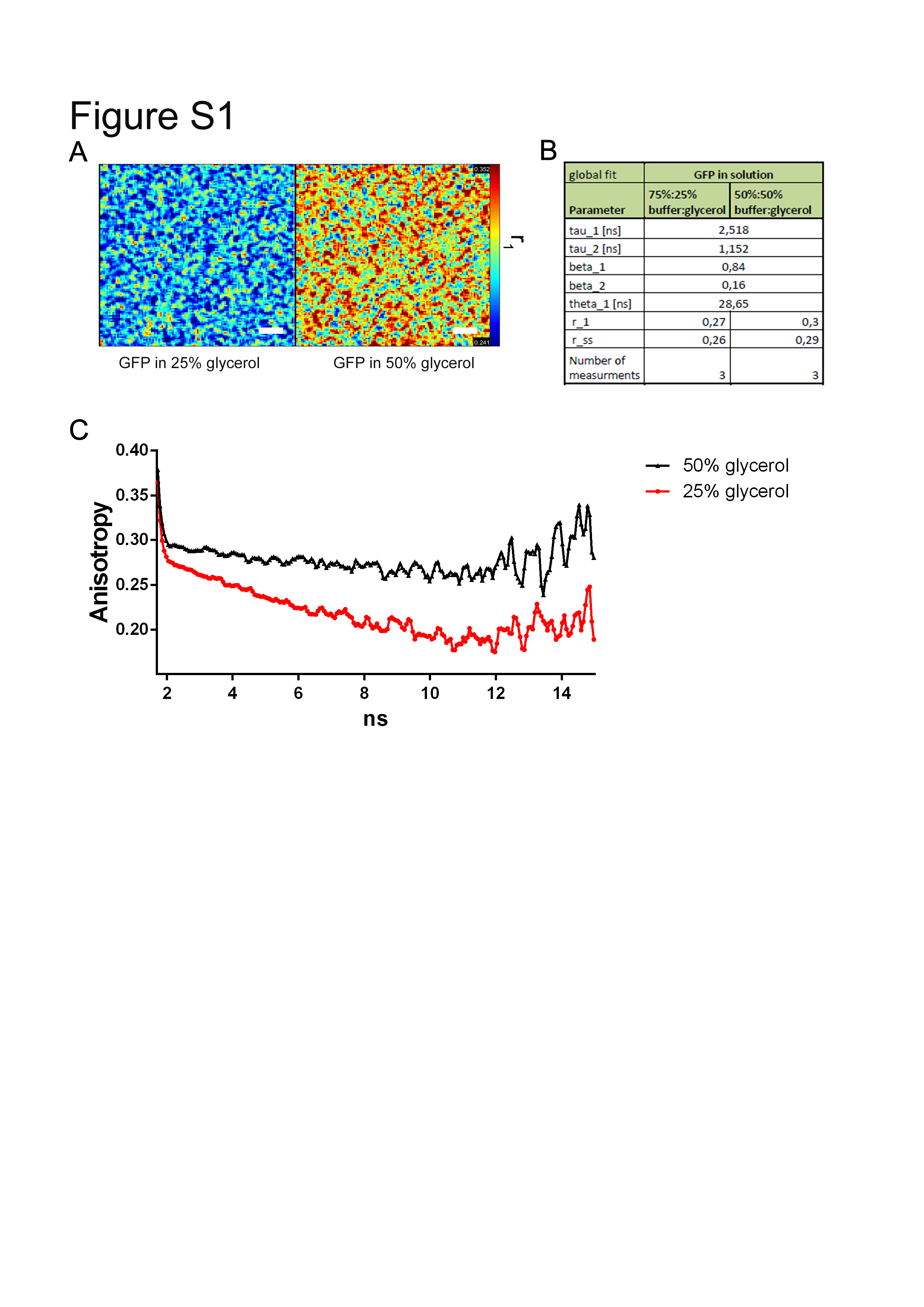

Supplement: Supplementary file 2 [file Image_1.TIF]

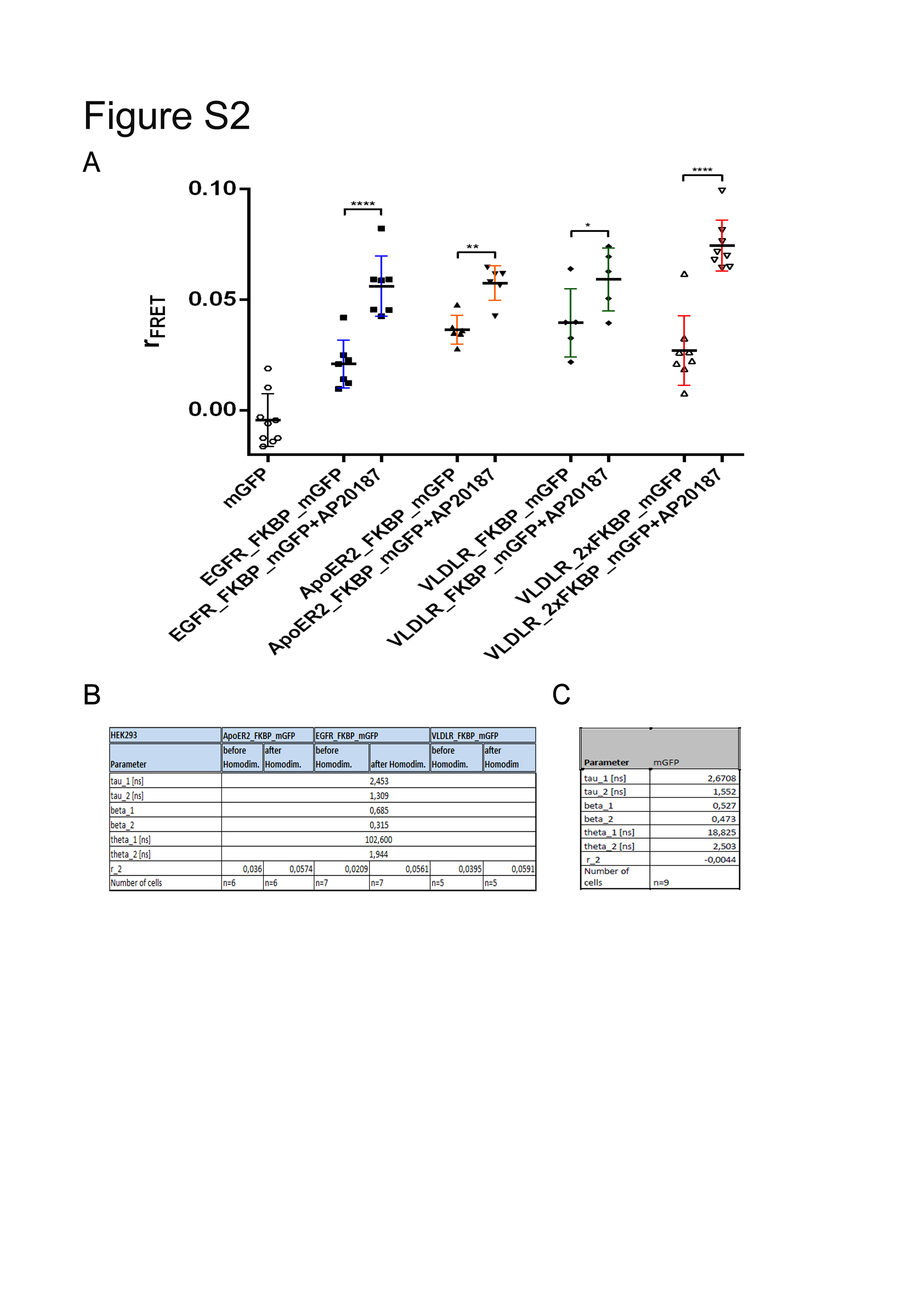

Supplement: Supplementary file 3 [file Image_2.TIF]

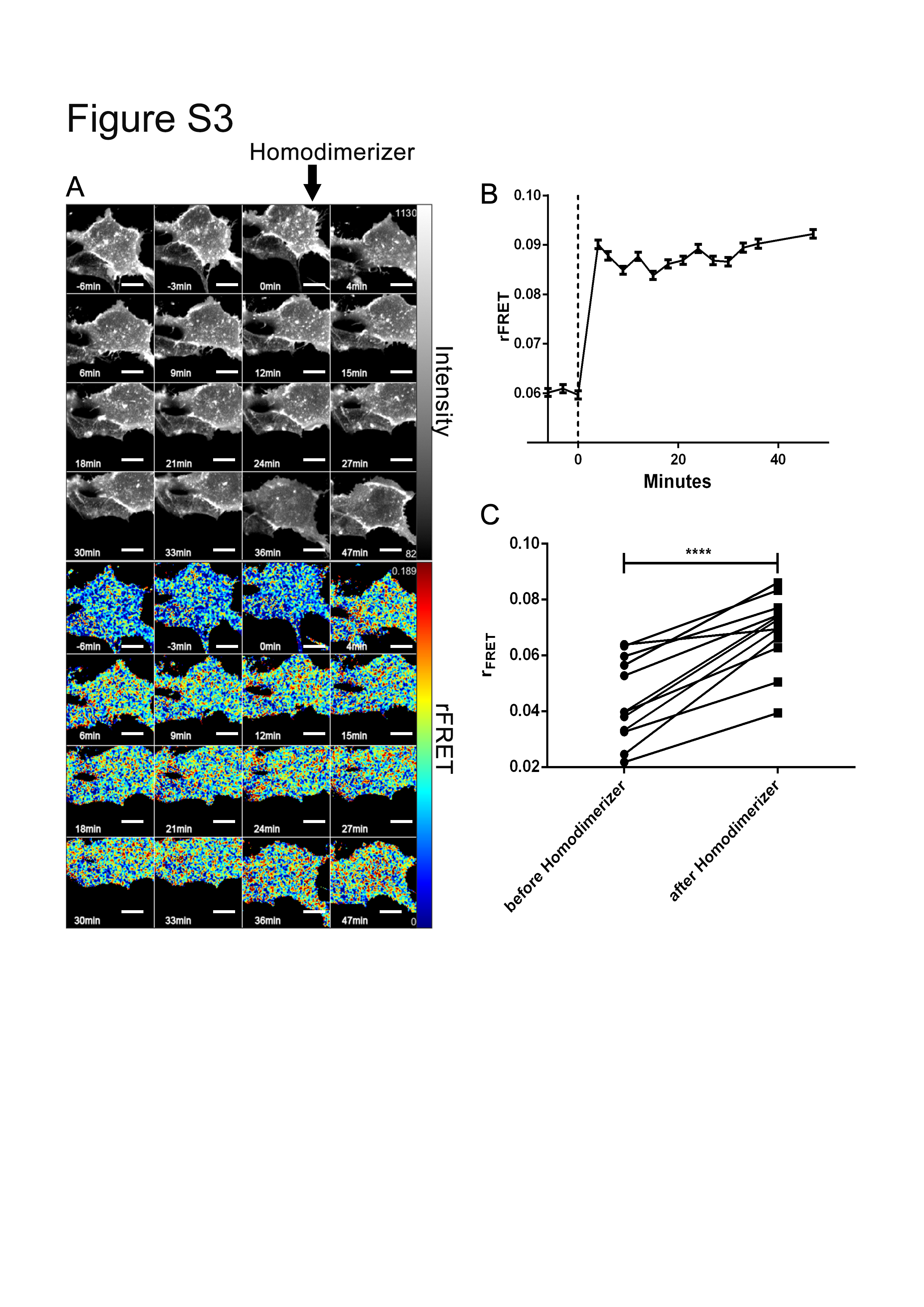

Supplement: Supplementary file 4 [file Image_3.TIF]

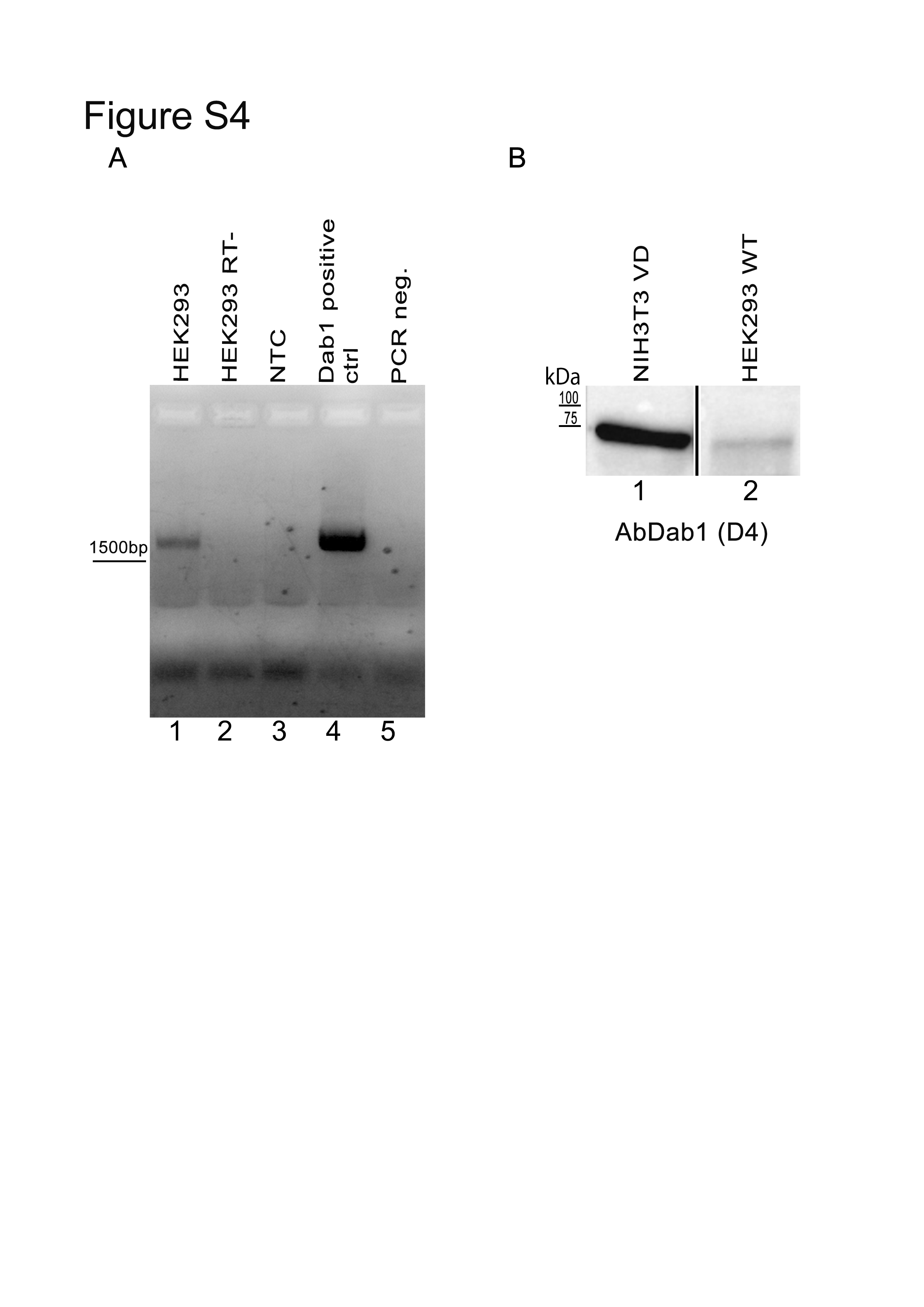

Supplement: Supplementary file 5 [file Image_4.TIF]
